# Ionic polarization-induced current–voltage hysteresis in CH_3_NH_3_PbX_3_ perovskite solar cells

**DOI:** 10.1038/ncomms10334

**Published:** 2016-02-08

**Authors:** Simone Meloni, Thomas Moehl, Wolfgang Tress, Marius Franckevičius, Michael Saliba, Yong Hui Lee, Peng Gao, Mohammad Khaja Nazeeruddin, Shaik Mohammed Zakeeruddin, Ursula Rothlisberger, Michael Graetzel

**Affiliations:** 1 Laboratoire de Chimie et Biochimie Computationnelles, ISIC, FSB-BCH, École Polytechnique Fédérale de Lausanne (EPFL), Lausanne CH-1015, Switzerland; 2 National Competence Center of Research (NCCR) MARVEL—Materials' Revolution: Computational Design and Discovery of Novel Materials, Lausanne CH-1015, Switzerland; 3 Laboratory of Photonics and Interfaces, ISIC, Swiss Federal Institute of Technology (EPFL), Lausanne CH-1015, Switzerland; 4 Group for Molecular Engineering of Functional Materials, ISIC-Valais, Swiss Federal Institute of Technology (EPFL), Lausanne CH-1015, Switzerland; 5 Center for Physical Sciences and Technology, Savanorių Avenue 231, Vilnius LT-02300, Lithuania

## Abstract

CH_3_NH_3_PbX_3_ (MAPbX_3_) perovskites have attracted considerable attention as absorber materials for solar light harvesting, reaching solar to power conversion efficiencies above 20%. In spite of the rapid evolution of the efficiencies, the understanding of basic properties of these semiconductors is still ongoing. One phenomenon with so far unclear origin is the so-called hysteresis in the current–voltage characteristics of these solar cells. Here we investigate the origin of this phenomenon with a combined experimental and computational approach. Experimentally the activation energy for the hysteretic process is determined and compared with the computational results. First-principles simulations show that the timescale for MA^+^ rotation excludes a MA-related ferroelectric effect as possible origin for the observed hysteresis. On the other hand, the computationally determined activation energies for halide ion (vacancy) migration are in excellent agreement with the experimentally determined values, suggesting that the migration of this species causes the observed hysteretic behaviour of these solar cells.

## Abstract (web summary)

The origin of hysteresis remains an open question in lead-halide perovskite solar cells. Here, Meloni *et al*. investigate the causes of hysteresis using an experimental and computational approach, finding that the observed hysteresis is due to halide ion-vacancy movement in the perovskite.

## Graphical abstract

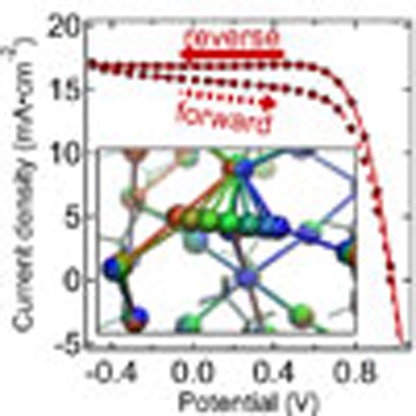

The perovskite MAPbI_3_ (methylammonium lead triiodide) and its halide analogues have emerged as one of the new and very interesting absorber materials for highly efficient solar cells^1^,^2^,^3^,^4^,^5^. Because of the ease of processing and wide range of applications (solar cells, photodetectors^6^,^7^,^8^,^9^,^10^ and lasing^11^,^12^,^13^,^14^) they have attracted intense attention in the research community. Currently, the increase of efficiency of the solar cell devices is one of the main focuses^15^,^16^, but the understanding of the basic operation principles of devices containing this material is also slowly evolving^17^,^18^,^19^. One of the main open questions is the origin of the observed hysteresis of the current-voltage (*JV*) curve of MAPbI_3_-based solar cells, first reported by Dualeh *et al*.^18^, and investigated in more detail by Snaith *et al*.^20^, Tress *et al*.^17^, O Regan *et al*.^21^ and other authors^22^,^23^,^24^. This effect complicates the determination of the ‘real' solar-to-electrical power conversion efficiency of such devices and can make ‘bad cells look good' as presented in a recent publication by Christians *et al*.^25^ Moreover, hypotheses were put forward suggesting a fundamental link between hysteresis and the limited long-term stability of halide perovskites^17^. Several ideas for the peculiar phenomenon underlying hysteresis have been proposed, the main advocated views involving either a ferroelectric effect^26^,^27^,^28^,^29^,^30^ or ionic (vacancy) movement^17^,^31^ inside the perovskite as probable cause.

Possible ferroelectric effects can be caused by the orientation of the organic (dipolar) cations, namely MA, or induced by deformation of the inorganic framework. In both cases the crystal acquires a net dipole moment. Under the effect of the external potential the MA molecules align with the external electric field, and the dipole moment of aligned MA molecules produces a balancing field lowering the effective field acting on the charge carriers. If the characteristic timescale necessary for the alignment is of the same order as the scanning time of the external potential, the effective potential acting on the charge carriers depends on the ‘history' of the experiment, resulting in the hysteresis. Although several theoretical^27^,^30^,^32^,^33^ and experimental^26^,^34^,^35^,^36^ studies have been undertaken in this direction, a recent investigation of Fan *et al*.^35^ showed that no room temperature ferroelectric behaviour could be measured.

An alternative hypothesis is that the hysteresis is due to the movement of ionic species^17^,^24^,^31^. It is well known from the literature that inorganic perovskites, like CsPbBr_3_ or CsPbCl_3_, are excellent halide conductors^37^,^38^. Moveable ions (or their vacancies) will induce a retarded reaction towards the change of electronic charge distribution of such a device under operation that could explain the so-called slow time component. Under the influence of an external biasing field or the ‘built-in' potential of the device, the migration of ionic vacancies may result in a net charge accumulation in certain regions of the MAPbX_3_ or at its contacts. The change of the concentration profile of the vacancies produces a balancing internal counterfield acting on the electronic charge carriers. Again, if the characteristic time of polarization of the sample is associated to the migration of ionic species is of the same order of magnitude as the potential scanning time, this phenomenon will result in a hysteresis of the *JV* curve.

Previous experimental studies^39^,^40^ have estimated the phenomenon at the origin of the observed hysteresis to take place on a timescale of microseconds to seconds. The relatively long timescale of this phenomenon suggests that it is a thermally activated process characterized by an activation energy sizably higher than the thermal energy available to the system. Here we have used a combined experimental and theoretical approach to determine this activation energy, and to identify the causative process. Experimentally we have determined the activation energy of the hysteretic process from the temperature-dependent measurement of the *JV* curve for MAPbI_3_ and MAPbBr_3_. By density functional based simulations we have determined the activation energy of different ion (vacancy) migrations in the crystal lattice as well as the characteristic rotational time of the MA ions. This combined approach allowed us to establish the general nature of the phenomenon and its activation energy. Our results support the hypothesis that hysteresis is due to halide ion (vacancy) migration induced polarization of the perovskite layer and exclude a ferroelectric effect due to the alignment of the MA ions.

## Results

### Experimental determination of the activation energy

**Figure 1:**
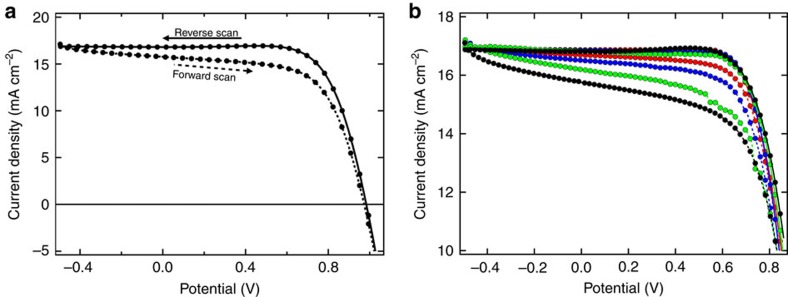
*JV* curves of an iodide-based device. (**a**) At 1 sun at −15°C (**b**) at different temperatures (for a better comparison all curves are scaled to reach the same *J*_sc_ for the reverse scans). The voltage scan rate is 50 mV s^−1^ between −0.5 to 1.1 V. Between each forward and backward scan the potential was kept constant for 50 s to let the device equilibrate (please see text for further explanation). Red (20 °C); blue (5 °C); green (−5 °C); and black (−15 °C).

To determine the activation energy, *E*_a_, of the process underlying the hysteretic behaviour, we have chosen a simple approach consisting in analysing the effect of the temperature on the *JV* curve of perovskite solar cell devices under illumination. A typical plot of different *JV* curves under illumination is presented in Fig. 1. The efficiencies of the devices with iodide were in the range of 10–14% PCE. Bromide devices had lower efficiencies, mainly because of the low *J*_sc_ (about 3–5 mA cm^−2^), resulting in a power conversion efficiency (PCE) of 2–4%.

The general protocol for most of the measurements was (if no other conditions are indicated): (i) 50 s waiting at reverse potential (−0.5 V); (ii) scanning the *JV* curve with 50 mV s^−1^ to 1.1 V forward bias (in the following denoted as ‘forward scan'); (iii) 50 s waiting at 1.1 V forward bias; and (iv) scanning the *JV* curve with 50 mV s^−1^ back to −0.5 V reverse bias (‘reverse scan').

The measurements have been performed at several different temperatures (normally between 20 and −20 °C). Figure 1b shows the dependence of the *JV* curve on the temperature. The measurements were started at 20 °C. After each measurement cycle, the *JV* curve of the device was re-measured at 20 °C to ensure that the device did not degrade during the measurements. Several features can be observed in the *JV* curve in Fig. 1b. The reverse scans show minor dependence on the temperature exhibiting generally a similar shape. Furthermore, one can observe a ‘bump' immediately before reaching a plateau of the current. This bump appeared for most of the devices though shape and change with temperature was not further investigated (please, see also text below regarding the phenomenon). The forward scans show a stronger dependence on the temperature. The slope of the overall current increases just as if the shunt resistance decreases with temperature. In other words, at room temperature the hysteresis between forward and reverse scan is small. Upon decreasing the temperature, the hysteresis between forward and reverse scan increases. To extract the *E*_a_, we measured the hysteresis as the difference of the current at a given voltage along the backward and forward scan, Δ*I*=*J*_B_(*V*)−*J*_F_(*V*), and studied its dependence on the temperature.

The simultaneous presence of several types of charge carriers (electrons, holes and ionic defects) in the system, the possibility that polarization affects the carrier dynamics in the perovksite layer or at the interface with the contacts, altering absorption, transport and recombination properties, makes it impossible to derive an analytical expression of the dependence of Δ*I* as a function of the temperature. A numerical solution, on the other hand, requires the determination of the dependence of the generation rate, diffusion coefficient, recombination rate and surface recombination velocity on temperature and the polarization of the perovskite layer. Moreover, in view of simulating hysteretic behaviour as a function of the temperature and sweeping rate, the solution of the time-dependent transport-reaction problem is required, and not the simpler steady-state solution of the time-independent problem. All this renders also the numerical solution option for interpreting experimental results out of reach at the moment. Thus, similarly to other authors (see, for example, Eames *et al*.^24^ and Yang *et al*.^41^), we have used an empirical relation between Δ*I* and *T*:


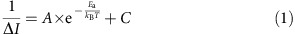


with A as prefactor, *k*_B_ as Boltzmann's constant, *T* as temperature and *C* as a constant. The reason for using the inverse of the difference between the backward and forward photocurrent is that this current difference is reduced when the process generating the hysteresis relaxes more quickly to the stationary condition during the voltage scanning. In other words, Δ*I* is expected to have an inverse proportionality to the ionic current (Δ*I*∼1/Δ*I*_ionic_), which depends on the corresponding diffusion coefficient, typically having an Arrhenius-like dependence on the temperature. Intuitive arguments supporting the empirical relation above between Δ*I* and *E*_a_ will be further elaborated in the Discussion section. In the following, we show that this relation is, indeed, obeyed by the experimental data. In addition, we will use results of atomistic simulations to show that the experimentally determined activation energy is, indeed, associated to a microscopic diffusion process.

**Figure 2:**
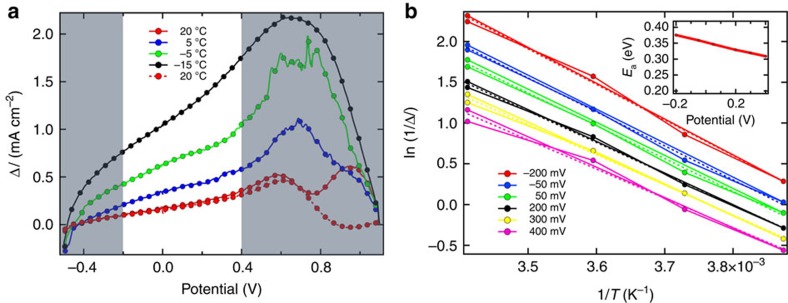
Δ*I* and potential dependent activation energies. (**a**) The current difference between forward and reverse voltage scan and the potential window (0.2–0.4 V) used for the fitting by the Arrhenius-type equation. (**b**) Plot of ln(1/Δ*I*) vs the inverse of the temperature for selected values of the potential (inset shows the determined activation energy independence of the potential).

Figure 2a shows Δ*I* as a function of the potential for an iodide-based device at different temperatures. Generally Δ*I* increases at lower temperature. One can also observe the strong increase of Δ*I* in the high forward bias region originating from the already mentioned ‘bump' often observed in the reverse scan of perovskite solar cells. In Fig. 2b ln(1/Δ*I*) is plotted against 1/*T* for selected potentials in the range of −0.2 to 0.4 V. By fitting these data with the Arrhenius-like equation, *E*_a_ is determined from the slope of ln(1/Δ*I*) vs 1/*T* (similar plots for the bromide-based devices are shown in Supplementary Fig. 1).

The activation energy determined at high forward (>0.4 V) and reverse (<−0.2 V) bias range showed the highest deviation from the *E*_a_ values at potentials in the medium range. This has two-fold reasons. One is the already mentioned bump in the reverse scan, as also observed by Tress *et al*. The origin of this bump is, presumably, the initially low field in the device for the reverse scan and, therefore, the low driving force for any kind of process which then sets in towards lower forward voltages. Therefore only the *E*_a_ values determined in the bias range −200 to 400 mV have been used for the calculation of the averaged *E*_a_ of Table 1. The hysteresis effect of the devices with bromide is generally less pronounced, which also leads to a higher error in the estimation of the activation energy from experimental data (*vide infra*). As a result, some Br-based devices did not seem to show the expected dependence on the temperature, for example, showing very-low or negative activation energies.

**Figure 3:**
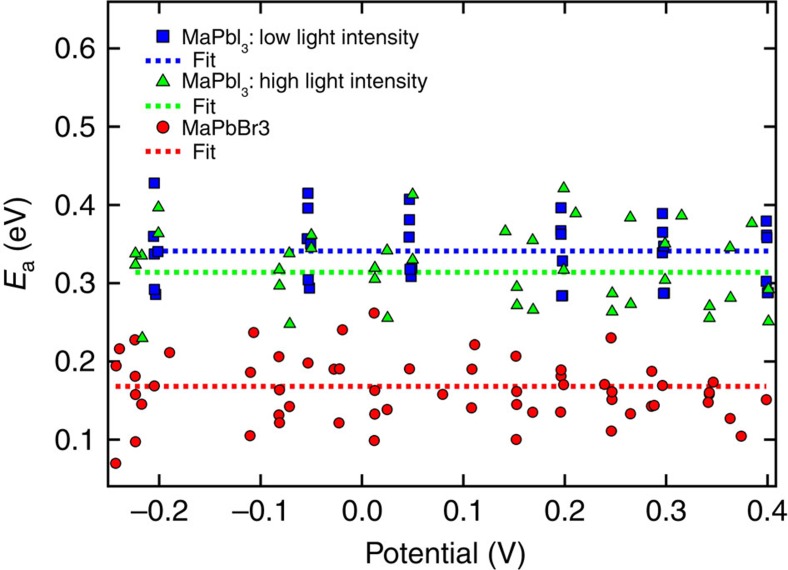
Potential dependent activation energies of different samples. Collection of the activation energies at different potential for the different samples measured.

The current density may also have an impact on the hysteretic behaviour. To compare the hysteresis at similar *J*_sc_ in the two halide systems we have measured the iodide-based devices also at low light intensities (0.1–0.2 sun, see Supplementary Fig. 2). We did not find any significant differences due to different light intensities in the *E*_a_'s shown in Fig. 3. In this figure, one can also clearly observe the lower activation energy for the devices with bromide. The different activation energies for the different devices with iodide (high and low light intensities) and with bromide are given in the Supplementary Fig. 3.

Δ*I* might also depend on the scan velocity and on the scan bounds of the voltage interval next to the already mentioned light intensity. It is clear that hysteresis can only be observed if the scan rates are performed within times similar to the characteristic timescale of the underlying phenomenon. In fact, it was shown by Tress *et al*. that if the scan velocities are too fast (⩾100,000 mV s^−1^) or too slow (<10 mV s^−1^), no hysteresis is observed^17^. Therefore, we have tested different measurement conditions—different scan rates (50, 100 and 200 mV s^−1^, see Supplementary Fig. 4a) and scan bounds (−0.5, −0.2 and 0 V, Supplementary Fig. 4b)—but could not observe any significant changes in *E*_a_.

Noticing that the major difference between the forward and backward scan of the *JV* curves at different temperatures is the slope of the *JV* curve along the forward scan, we also computed the activation energy associated to this resistance-like term at 0 V. The barriers obtained from Δ*I* and the slope (in principle equal to an Arrhenius-type relation of ln(1/*R*) vs 1/*T*) are consistent (see Supplementary Figs 1d, 2d and 5a). This justifies the approach of taking either Δ*I* or the slope to measure the activation energy of the slow process as hysteresis in this voltage range is mainly governed by a rate limited process that does not strongly depend on the actual voltage applied during the scan. Thus, the determined *E*_a_ directly reflects the activation energy of this slow process, which is reacting retarded to the change of the applied voltage (as will be explained in more detail in the discussion section and in the gedankenexperiments in the last part of the SI).

Extracting the activation energies for the different devices by the procedure(s) described yields a clear trend (see Supplementary Figs 3 and 6). The iodide-based devices show activation energies of in average 333±47 meV, and the bromide-based devices of about 168±43 meV. When using the determination over the slope of the forward scan (at 0 V), the tendency is similar with *E*_a_ being 275±19 meV for the iodide-based perovskite devices and 176±43 meV for the bromide-based perovskite devices (see Supplementary Fig. 5b). As mentioned above, the relative error on the estimation of *E*_a_ is larger for the bromide devices but the tendency is clear. The *E*_a_ dependence on the halide rules out that *E*_a_ describes a temperature-activated transport in the contacting materials, which are the same for all devices. In addition, changed transport properties of the contacting materials should take effect as series resistance under high forward bias reducing the fill factor. The results reported above clearly indicate that the nature of the halide significantly affects the hysteresis via the barrier of the associated thermally activated process, which is lower for bromide than for iodide. This suggests that the process underlying hysteresis involves movements of halide ions (or their vacancies).

### Simulations of the ferroelectric effect

Present experiments show that hysteresis is due to a ‘thermally activated' process, with an associated barrier in the range of ∼0.1–0.4 eV, depending on the type of perovskite used in the device and the conditions of the experiment. To identify what is the microscopic process causing hysteresis, in particular, whether it is due to ferroelectricity or ionic polarization, we performed two types of simulations. First, we performed ∼30 ps long first-principles (on the basis of density functional theory) molecular dynamics simulations (MD) at various temperatures (*T*=100, 200, 300 and 400 K) starting from the tetragonal MAPbI_3_ crystal phase. The computational sample consisted of a 2 × 2 × 2 supercell of the simple tetragonal analogue of the experimental body-centered tetragonal crystal^42^, containing 32 stoichiometric units (384 atoms).

The system equilibrated at different temperatures is able to assume different crystalline phases with a trend consistent with experimental results^42^,^43^. At 100 and 200 K, the atoms arrange in an orthorhombic-like phase with non-negligible values of all the three tilting angles. At 300 K, two of the three tilting angles are reduced and the structure becomes tetragonal-like. Finally at 400 K, all the three tilting angles are approximately 0° and the structure is cubic-like. More details on the temperature-dependent simulations are also provided in the Supplementary Notes 1 (Supplementary Figs 7–12).

Albeit the relatively short simulation times of 30 ps, the computational results suggest that a ferroelectric origin of hysteresis in unlikely. Hysteresis induced by ferroelectricity might be due to either a break of symmetry of the PbI_3_ lattice, perhaps induced or enhanced by the lack of inversion symmetry in the crystal due to the MA cation, or by the alignment of the polar MA molecules^27^,^44^. A break of symmetry in the PbI_3_ lattice should result in a histogram of the Pb–I bond distances (<3.2 Å) with multiple maxima. The computed distribution (*g*_PbI_(*r*), Supplementary Fig. 7) shows no evidence of this feature at any of the temperatures investigated, suggesting that there is no break of symmetry in the PbI_3_ framework whatever the crystal phase of the sample is.

**Figure 4:**
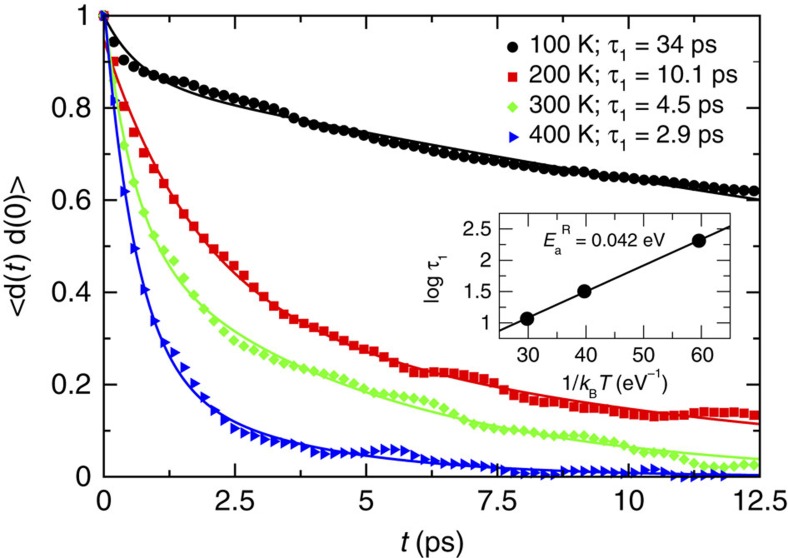
Time-dependent autocorrelation function of the unit C–N vectors. The autocorrelation function has been computed averaging over the microcanonical ensemble sampled by constant number of particles, volume and energy (NVE) first-principles MD simulations performed at four different energies corresponding to average temperatures of 100, 200, 300 and 400 K. In the inset log *τ*_1_ vs 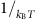 is shown, together with the linear fitting from which the rotational reorientation activation energy is obtained.

Furthermore, we investigated the possibility that ferroelectricity originates from a persistent preferential alignment of MA ions. Under the effect of the external bias plus built-in potential MA molecules might align with the overall electric field and produce a counterfield. To determine the characteristic rotational reorientation time, the time correlation function of the C–N unit vector, 〈***d***(*t*)·***d***(0)〉 (***d***=***r***_*N*_−***r***_*C*_/|***r***_*N*_−***r***_*C*_|), is computed, and it is fitted with a double exponential decay, 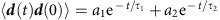. The time for MA molecules to loose memory of their initial orientation, *τ*_1,_ is on the picoseconds timescale at 200–400 K (see Fig. 4). At 100 K the reorientation time is much longer, probably because of the fact that complete rotation of MA ions is hindered at this temperature. These results are consistent with previous first-principles calculation using a different, more qualitative, approach to determine the characteristic reorientation time^39^,^45^, recent classical MD simulations^46^ and experimental data^47^,^48^,^49^,^50^. The consistency with classical MD results on bigger samples suggest that no relevant finite size effects affect the estimated reorientation times. From the dependence of *τ*_1_ on the temperature in the range 200–400 K, we estimated an activation energy for MA reorientation of 0.042 eV (inset of Fig. 4), consistent with experimental results^50^.

If the dynamics of the MA molecules is uncorrelated, that is, if they rotate independently from each other, the characteristic orientational correlation time determined in the simulations is also the time the sample takes to polarize under the action of a bias. This leads to the conclusion that polarization due to dipole alignment takes place on the picosecond timescale. This is too short a time to account for the *JV* hysteresis, that is associated to a process with a characteristic time in the milliseconds-to-seconds range^39^,^51^. To estimate the amount of correlation between MA molecules, we computed the spatial correlation function, 〈***d***_***i***_·***d***_***j***_〉 with ***d***_***i***_ and ***d***_***j***_ unit C-N vectors of molecules *i* and *j* (Supplementary Fig. 12). The spatial correlation function is 1 if two MA ions have the same orientation during the simulation, −1 if they have opposite orientation and 0 if the orientation of one is independent of that of the others. MD simulations results show that at room temperature the correlation between MA ions is small. This, indeed, is consistent with the fact that all the MA ions undergo a quick decorrelation, as shown by the time autocorrelation curves of individual molecules, 〈***d***_***i***_(*t*)·***d***_***i***_(0)〉 (see Supplementary Fig. 10). Thus, we expect that the alignment of the sample takes place on the timescale of the rotational reorientation time of a single MA ion, that is, on the picosecond timescale estimated above. Further information about these simulations are available in the SI (Supplementary Figs 9, 10 and 11).

Recent Monte Carlo simulations^32^ on a lattice model of MAPbI_3_ have estimated the alignment under the action of an electric field to take place in 10^4^ spin steps. Making an accurate estimation of the reorientation time from Monte Carlo steps is not simple, as there is no one-to-one correspondence between a Monte Carlo step per MA molecule and the time MA ions would take to cover the corresponding reorientation. However, an approximate upper limit estimation of the polarization time of the sample can be obtained by assuming that a global Monte Carlo step, that is, one step for each molecule in the sample, costs a time corresponding to the rotational reorientation time, *τ*_1_. This would yield an alignment time of ∼50 ns (10^4^ steps × ∼5 ps), still too short for MA-reorientation-related ferroelectricity to be responsible for hysteresis.

### Simulations of the ionic migration

To probe the second hypothesis, that is, that a bias-induced stepwise migration of ions might result in a change of their local concentration and then in a balancing internal counterfield, we performed MD simulations on systems containing a single MA^+^, Pb^2+^ or I^−^ vacancy, respectively. The reason for focusing on vacancies is that previous experiments have shown that ionic transport in Br and Cl perovskites is most likely assisted by this type of defect.

During the 10 ps of first-principles MD, no spontaneous ionic vacancy migration was observed. This is not surprising as it is known that ionic/vacancy migration in MAPbX_3_-related materials is slower than the timescale of our simulations^37^,^52^,^53^. The longer timescale of ionic migration processes makes this second phenomenon a more plausible candidate as source of the observed *JV* hysteresis. To assess this, we performed string simulations^54^ aimed at computing the vacancy-driven ionic migration path and the associated energy barrier, *E*_a_, for MAPbI_3_ and MAPbBr_3_. In the case of MAPbBr_3_, for which the stable phase at room temperature is cubic, we considered both tetragonal and cubic structures. This allows to distinguish between the effect of halide substitution and phase change on the migration barriers. In the case of cubic MAPbBr_3_ we observed no qualitative changes with respect to the tetragonal case, and the activations energies typically are between the values estimated for the corresponding tetragonal system.

**Figure 5:**
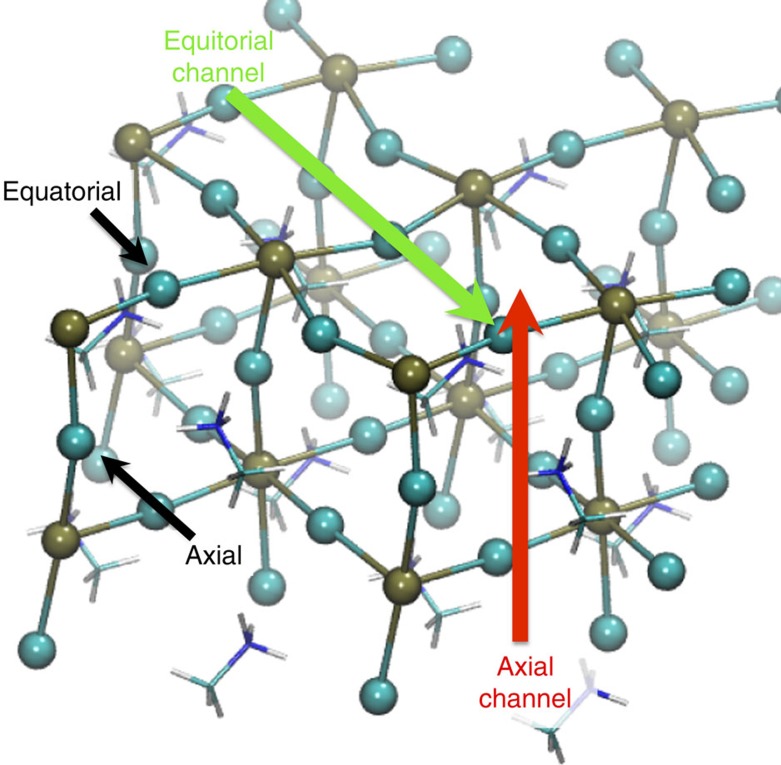
Propagation channels. Periodic crystalline (defect free) MAPbI_3_ sample. Cyan and brown spheres represent I and Pb ions, respectively. MA is shown as sticks. Periodic boundary conditions are applied in all the crystallographic directions. Black arrows point to equatorial and axial I^−^ ions. Red and green arrows indicate the axial and equatorial channels for MA^+^ and Pb^2+^ ion migration.

**Figure 6:**
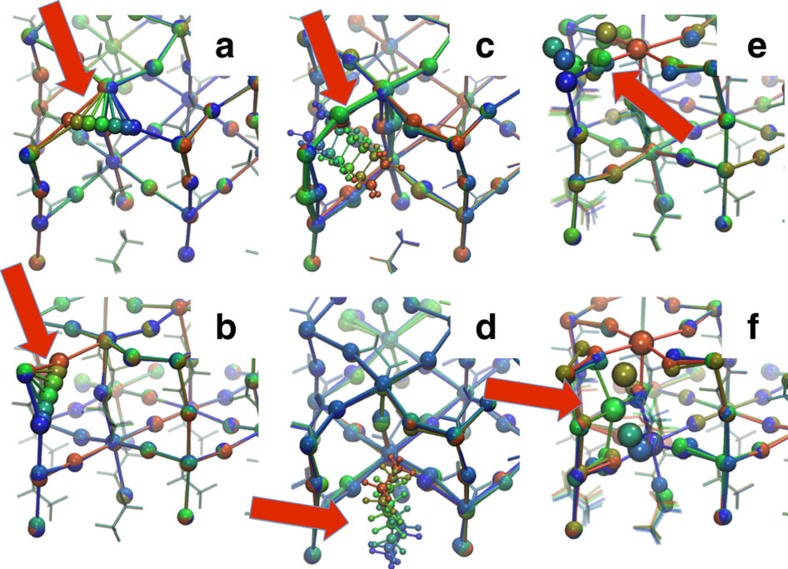
Stroboscopic images of the ionic migration paths in MAPbI_3_. (**a**–**f**) Paths for the corresponding migration events in MAPbBr_3_ are analogous. The colours of the frames go from blue (initial states) to green (intermediate states) to red (final states). (**a**,**b**) Migration path of I^−^ along the equatorial-to-equatorial and equatorial-to-axial channels, respectively. (**c**,**d**) Migration paths of MA^+^ along the equatorial and axial channels, respectively. (**e**,**f**) Migration paths of Pb^2+^ along the axial and equatorial channels. Both paths are rather complicated. The details are described in the main text.

Moreover, to estimate the possible effect on *E*_a_ arising from the arbitrary choice of the initial orientation of MA, with its high orientational mobility, we also investigate two systems containing the spherically isotropic Cs^+^ monovalent cation, namely CsPbI_3_ and CsPbBr_3_. For these systems we considered the tetragonal phase. Because of the crystal symmetry, MA^+^/Cs^+^ and Pb^2+^ ionic migration can take place either along or perpendicularly to the tetragonal axis, and the barriers of these two processes can differ. Here we consider both cases: axial (a) and equatorial (e) migration (see Fig. 5). It is also important to remark that tetragonal perovskite crystals contain two non-equivalent I/Br sites. The first, denominated axial in the following, is the one in which the halide ion forms the Pb–X–Pb triplex oriented along the tetragonal axis (see Fig. 5). The second, the equatorial site, is the one in which the halide forms Pb–X–Pb laying on the plane orthogonal to the tetragonal axis. Halide migration can take place from an equatorial to another equatorial site, e2e, or from an equatorial to an axial site, e2a (or vice versa, a2e). Stroboscopic images of the migration processes mentioned above for the case of MAPbI_3_ are shown in Fig. 6a–f.

The migration barriers for all the processes and systems mentioned above are reported in Table 2. The comparison of the migration paths of the X^−^, Pb^2+^ and A^+^ ions easily explains the difference in the migration energy of the various species. Figure 6a,b shows that a very-small distortion of the crystal structure accompanies halide migration, while migration of A^+^ (Fig. 6c,d) and Pb^2+^ (Fig. 6e,f) requires a significant even though local rearrangement of the crystal structure.

The migration of halide ions essentially affects only the PbX_6_ units involved in the event, with negligible distortions of the rest of the lattice. The migration barrier does not change significantly replacing MA^+^ with Cs^+^. In particular, the trend of the migration barrier with the chemical nature of the halide is confirmed. This suggests that our results for MAPbX_3_ samples are not significantly biased by the arbitrary choice of the initial orientation of the MA ions. The axial vacancy is significantly less stable than the equatorial one (Δ*E*∼0.1–0.2 eV), and a vacancy in the axial state is expected to migrate towards an equatorial one. Thus, a complete migration event along the axial channel, bringing the vacancy from an equatorial site to another, requires two steps: an e2a migration followed by an a2e one. Thus, this channel requires the crossing of two barriers. In the case of MA-perovskites, the first barrier, e2a (450–460 meV), is sizably higher than the single barrier of the equatorial channel, e2e (200–280 meV), and the one-step e2e migration channel will give the major contribution to the halide transport in perovskites. It is, then, the activation energy of this channel determining the halide transport in perovskites.

The migration of the monovalent cation requires the opening of the PbX_3_ framework separating the initial and final A^+^ sites. The shape of this framework is different in the axial and equatorial directions. Thus, the activation energy and the migration path, including the orientation of the MA ions along it, depend on the channel, whether equatorial or axial. Given the high orientation disorder of MA^+^, it is expected that under experimental conditions this ion migrates following paths characterized by different orientations of the C-N bond and, possibly, different migration barriers. We expect that the migration barrier of Cs^+^ represents a lower bound for that of MA^+^, consistent with the fact that Cs^+^ is smaller than MA^+^(as suggested by the larger size of the lattice of MA-perovskites with respect to Cs-perovskites computed in simulations).

Also the migration of Pb^2+^ requires a significant distortion of the crystal structure. This is necessary to let the Pb^2+^ ion leave its original site and enter into the new one. This explains the high barrier for the migration of Pb^2+^.

Walsh *et al*.^55^ have shown that the defects in MAPbI_3_ are formed according to the Schottky mechanism, in which the amount of iodide and cation defect concentrations have the same order of magnitude. In particular, according to Walsh *et al*. the most probable point defect formation reaction is


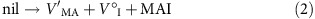


with an associated formation enthalpy of 0.08 eV. These results, taken together with the calculated activation barriers for defect migration, show that not only *V*°_I_ is the most mobile defect, but it is also present in similar concentrations with the other defects considered in the present work, and that, indeed, the ionic mobility is the limiting step in the vacancy-driven polarization of the perovskite sample. Thus, considering the migration barriers of all the ionic species shown in Table 2, at room temperature the timescale of the migration of the MA^+^ and Pb^2+^ ions is 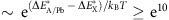 larger than the one for X^−^ ions. This is consistent with the experiments by Yang *et al*.^41^ with solid-state electrochemical cells of MAPbI_3_, in which it has been shown that the active mobile ion is iodide.

Indeed, the I^−^ and Br^−^ computational migration barriers along the e2e channel are in very good agreement with the experimental activation energies. In addition, the effect of halide substitution follows the experimental trend discussed in the previous section, with a lower barrier for halide migration in Br than in I perovskites. The good match between experimental and computational results, both in the absolute value of the migration barriers and in the trend with halogen substitution, together with experimental results reported previously^17^,^39^, strongly support the hypothesis that hysteresis is due to the polarization of the perovskite layer associated to halide-vacancy migration. As proposed in refs 17, 39, halide ions/vacancies migrate in the same direction as the corresponding charge carriers. Since ions are not extracted at the contacts, they accumulate at the interface of the electrodes producing a balancing potential that reduces the efficiency of collection of charge carriers. In extreme cases, and in the absence of the compact-TiO_2_ hole-blocking layer, the internal balancing potential can significantly reduce the *V*_oc_ (ref. 17).

## Discussion

Before discussing experimental and computational results, it is worth giving the intuitive arguments at the basis of the empirical relation between Δ*I* and *T* (Eq. (1)). A first observation is that polarization of the sample due to ionic movements, or to the alignment of MA molecules, produces a counterfield opposing to the external bias plus built-in voltage. This results in a reduction of the measured current. A possible explanation is that reducing the overall field for extracting charge carriers increases electron–hole recombination. A second observation is that in this work we focus on a temperature range, in which hysteresis shrinks with the temperature (see below). In this case, the faster is the relaxation to the stationary condition from the preset state along the voltage scanning, the lower is the hysteresis. Thus, we assume that hysteresis is in inverse proportion to the relaxation rate. The relaxation rate is related to the diffusion coefficient, in the case of ionic polarization, or the reorientation time, in the case of ferroelectric polarization. In both cases, the dependence on the temperature is via an Arrhenius-like relation characterized by an activation energy. These arguments are summarized in Eq. (1). The values determined for the activation energy in our experiments are in close agreement with the values reported by several other authors using different experimental approaches^24^,^37^,^38^,^41^,^56^, supporting the validity of such an empirical Δ*I*−*E*_a_ relation.

The comparison between experimental and theoretical results suggests that hysteresis is due to ionic movements rather than ferroelectricity. Here we propose a possible mechanism explaining the experimental observations based on this hypothesis. The effect of ionic displacement is the polarization of the perovskite layer at the contacts, which eventually changes the characteristic properties of the device. A similar effect has been exploited in the field of piezophototronics^57^,^58^. It has been shown that the accumulation of cations or anions at the interface of, for example, p–n junctions can significantly alter the band bending and can also affect the conduction and valence band edge positions of the involved semiconductors (or work functions of the contacting materials). In perovskite-based solar cells, mobile ions can similarly accumulate at the interface with the contacting materials under the action of the electric field generated by the space charge region at the contacts. The effect is minimized close to *V*_oc_, where the built-in potential is approximately balanced by the external bias and a minimum force is acting on the ions. Thus we can assume that at *V*_oc_ ions are distributed almost uniformly in the perovskite layer. In contrast, when the external bias is zero (and the internal field is high), ions accumulate at the contact(s). The switching from the polarized to unpolarized state is not instantaneous, and this results into the hysteresis.

However, considering a broader temperature range, the dependence of the hysteresis with temperature is more complex: In the temperature regime below ∼180 K, an increase in the hysteresis can be observed as shown by Zhang *et al*.^59^ Since the perovskite is in its ferroelectric crystal phase, with the *J*_sc_ being very low and the overall solar cell efficiency going under 0.1% the reasons for the hysteretic behaviour are more complex and its interpretation goes beyond the scope of this manuscript. At 180 K, nearly no hysteresis is visible in the *JV* curve. At this temperature the ions do not move significantly on the timescale of the scanning plus dwell time (∼60–80 s, depending on the scanning rate). In absence of polarization, hysteresis cannot be observed as the state of the system at the given voltage is independent of scan direction. Increasing the temperature increases the mobility of ions and, thus, the polarization during the dwell time at the starting point of the voltage scanning. During the voltage scan, which lasts for maximum ∼30 s in the case of the slowest scan rate (50 mV s^−1^), the system cannot reach the stationary condition at each voltage and hysteresis increases with *T* (in the temperature regime with 180 K<*T*<∼240 K). There exists a temperature, *T*>∼240 K, at which the system is able to approach a stable polarization during the dwell time. Thus, any further increase of the temperature does not significantly increase the initial polarization before the scan. However, mobility keeps increasing with the temperature and the system can more closely approach the voltage-dependent stationary ionic distribution during the scan. This results in a reduction of the hysteresis. This behaviour is shown in Supplementary Figs 1a and 2a, which show a slight decrease of the measured current with increasing temperature in the backward scan, and a complementary increase in the forward scan. Summarizing, a complex dependence of the hysteresis on temperature is observed, and this is due to the interplay of the effect of the increased mobility of the ions on the polarization during the dwell time at the starting points of the scanning, and the relaxation during the scanning. The effect of the temperature between 80 and 360 K has been investigated in more detail by Zhang *et al*.^59^

The outlined model leading to the observed hysteresis can explain the characteristics of perovskites solar cells measured in this and other recent works. Tress *et al*.^17^ have observed a shift in the forward and backward *JV* curves in the dark by changing the pre-conditioning potentials. This device was made without a TiO_2_ blocking layer. In this case, the charge polarization can act as a term balancing the built-in potential and/or changing the work function of the fluorine-doped tin oxide (FTO) due to the different ionic distribution at this interface, and, thus, shifting the dark current curves. Almora *et al*.^60^ have reported *JV* curves in the dark presenting a capacitive loop. The (retarded) variation of ionic polarization during the voltage scans can result into a change in the extension or charging of the space charge zone, which is equivalent to a varying capacitance along the backward and forward scan. Our experiments and calculations, as well as the above mentioned experiments, interpreted in light of the model described here, suggest that the *JV* characteristics of perovskite solar cells under illumination are strongly influenced through a modulation of the current by ionic polarization. We have made two gedankenexperiments that are presented in the Supplementary Notes 2 highlighting in more detail the thoughts we have presented in this paragraph (see also Supplementary Fig. 13).

The possibility that the width of the space charge region, edge positions of the semiconductor bands (which also includes the contacting semiconductors, for example, the TiO_2_) or the work function of the conductive substrate (for example, FTO as shown by Tress *et al*.) are altered by changes of the ionic environment, leads to a complicated interplay of the different materials. We want to stress that the qualitative model outlined above and in the SI requires additional investigation to identify the detailed effect of ion accumulation on band bending and band edge position, and ultimately on charge separation and collection.

In summary, here we have shown that the hysteresis observed in *JV* curves of different MAPbX_3_ perovskite solar cells is due to a thermally activated process and we have determined the associated activation energy. The variation in the processing parameters for the preparation of the perovskite layer, as well as different measurement conditions (*JV*-scan rate and value of the potential at which hysteresis is measured) did not significantly affect the determined values for the activation energy, demonstrating the independence of the origin of the hysteretic effect on the processing and measurement conditions. Consistently, the activation energy for the bromide- and iodide-based devices showed an average of 168±43 and 333±47 meV, respectively.

We paralleled the experimental investigation with first-principles MD simulations to determine the characteristic time for (re-)orienting MA molecules. We could show that this characteristic time, in the picosecond range, is too short for the process being associated with *JV* hysteresis. Furthermore, we determined the activation energy of the migration of vacancies of the various ionic species forming the perovskite and show that the lowest activation energy for vacancy migration is the one for halides. Its values matches well with the experimentally determined activation barriers involved in hysteresis. The dependence of this barrier on the type of halide computed in the simulations is also in agreement with the experimental trend. Present experimental and computational results strongly support the hypothesis put forward in ref. 17 that hysteresis is due to polarization of ionic charges in the perovskite layer under the influence of the built-in and applied potential. The mobility of the other possible ionic species (MA^+^ and Pb^2+^) than the halides is much lower, and we do not expect them to give any significant contribution in the polarization of devices in experiments with a scanning rate in the range of 10 to 10,000 mV s^−1^.

## Methods

### General methodological information

Refer to the Supplementary Information for experimental section, figures on *E*_a_ for different scan velocity and scan bounds, different preparation methods and light intensities. Additional computational details and results, and further discussion of the outlined model is available free of charge via the Internet at http://pubs.acs.org.

## Additional information

**How to cite this article:** Meloni, S. *et al*. Ionic polarization-induced current-voltage hysteresis in CH_3_NH_3_PbX_3_ perovskite solar cells. *Nat. Commun.* 7:10334 doi: 10.1038/ncomms10334 (2016).

## Supplementary Material

Supplementary Information Supplementary Figures 1–13, Supplementary Notes 1 & 2, Supplementary Methods and Supplementary References.

## Figures and Tables

**Table 1:** Averaged activation energies.

**Perovskite**	**Light intensity**	***E***_**a**_ **(meV)**
MAPbI_3_	High (∼1 sun)	314 (±48)
	Low (0.1–0.2 sun)	341 (±42)
	All iodide devices	333 (±47)
MAPbBr_3_	∼1sun	168 (±43)

Determined average activation energies for iodide and bromide-based MAPbX_3_ devices. Values averaged between −200 and 400 mV.

**Table 2:** Activation barriers for ionic migration.

	***E***_**a**_ **(meV)**	
	**A**^**+**^**=Cs**^**+**^	**A**^**+**^**=MA**^**+**^
*APbI3*		
Vacancy I^−^		
e2e	360	**280**
e2a	290 (170)	450 (130)
Vacancy A^+^		
e	590	1,120 (880)
a	1,160	700 (600)
Vacancy Pb^2+^		
e	810	1,390
a	990	1,780

		**Tetragonal**	**Cubic**
*APbBr3*			
Vacancy Br^−^			
e2e	270	**200**	**220**
e2a	290 (160)	460 (90)	
Vacancy A^+^			
e	700	1,130 (950)	1,010
a	1,200	800 (700)	
Vacancy Pb^2+^			
e	940	1,350	1,620
a	1,220	1,800	

In the tetragonal phases, there are different possible migration channels. For the e2a case of halide migration, where the initial and final states have a different energy, the barriers of the inverse process, a2e, are reported in parentheses. The e2e halide migration channel (reported in bold in the table) is expected to be the most efficient ionic transport path, and it is the corresponding activation energy that must be compared with the experimental values (168 meV for MAPbBr_3_ and 333 meV for MAPbI_3_). The initial and final states of the MA^+^ migration are non-equivalent, due to non-equivalent MA orientation in the initial and final states. Also in this case we report in parentheses the activation energy of the reverse process.
