# Supplementary material for: Ionic polarization-induced current–voltage hysteresis in CH3NH3PbX3 perovskite solar cells
Source: Nat Commun. 2016 Feb 8;7:10334. doi: 10.1038/ncomms10334 (PMC4748116; doi:10.1038/ncomms10334)
Supplement: Supplementary Information — Supplementary Figures 1–13, Supplementary Notes 1 & 2, Supplementary Methods and Supplementary References. [file ncomms10334-s1.pdf]

Supplementary Information

Supplementary Figures

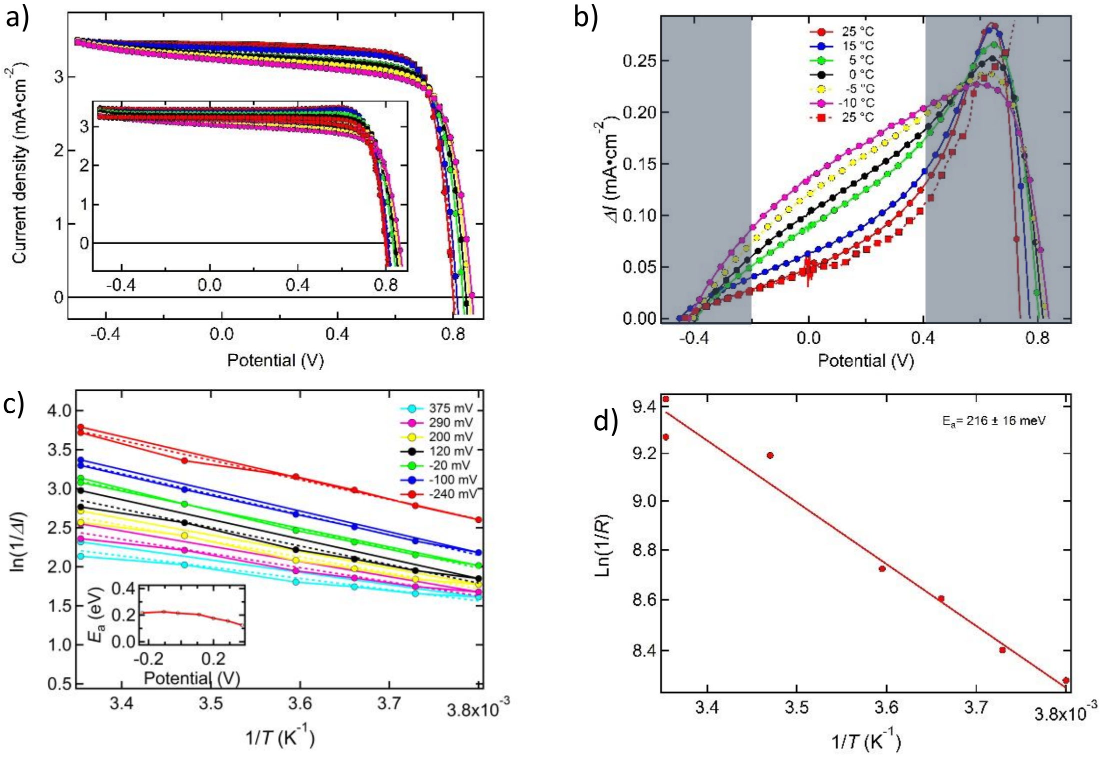

Supplementary Figure 1. a) *JV* curves of a bromide device (only forward scan and set to similar J_{sc}). Inset shows the forward and reverse scan as measured. b) ΔI plotted against the potential. c) Plot of ΔI vs 1/*T* for potentials between -0.2 and 0.4 V. Inset shows the activation energy determined at the potentials. d) Plot of the ln(1/*R*) from the slope at 0V versus 1/*T*.

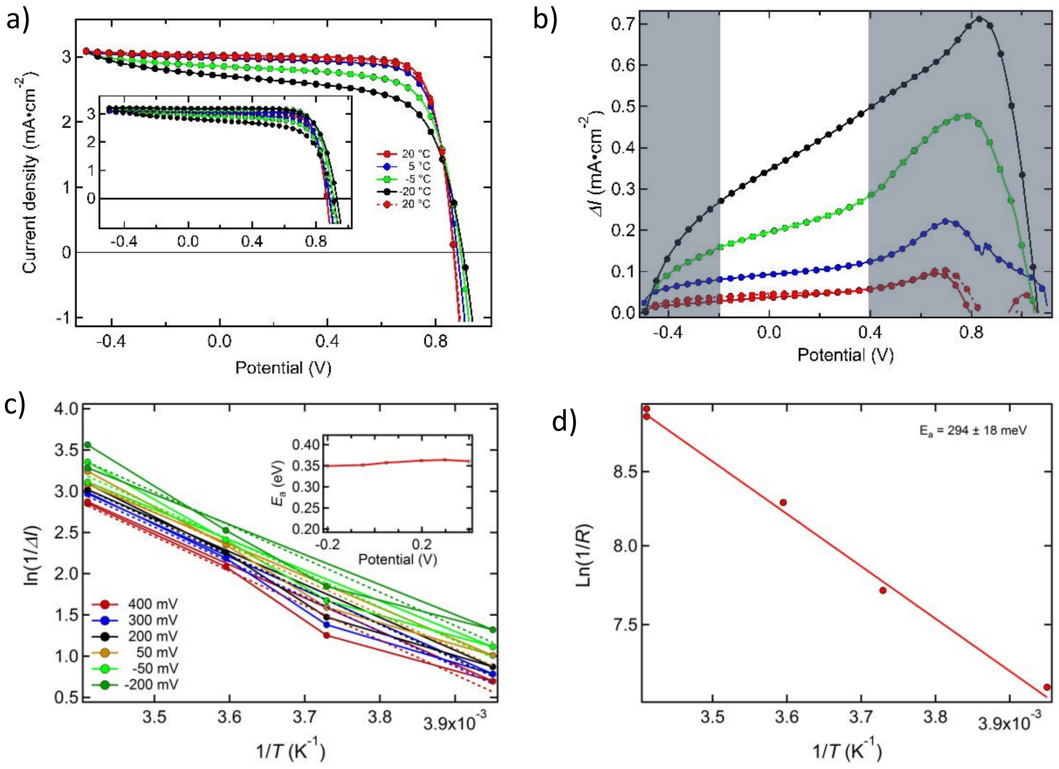

Supplementary Figure 2 a) *JV* curves of an iodide device at low light intensity (only forward scan and set to similar J_{sc}). Inset shows the forward and reverse scan as measured. b) ΔI plotted against the potential. c) Plot of ΔI vs 1/*T* for potentials between -0.2 and 0.4 V. Inset shows the activation energy determined. d) Plot of the ln(1/*R*) from the slope at 0V versus 1/*T*.

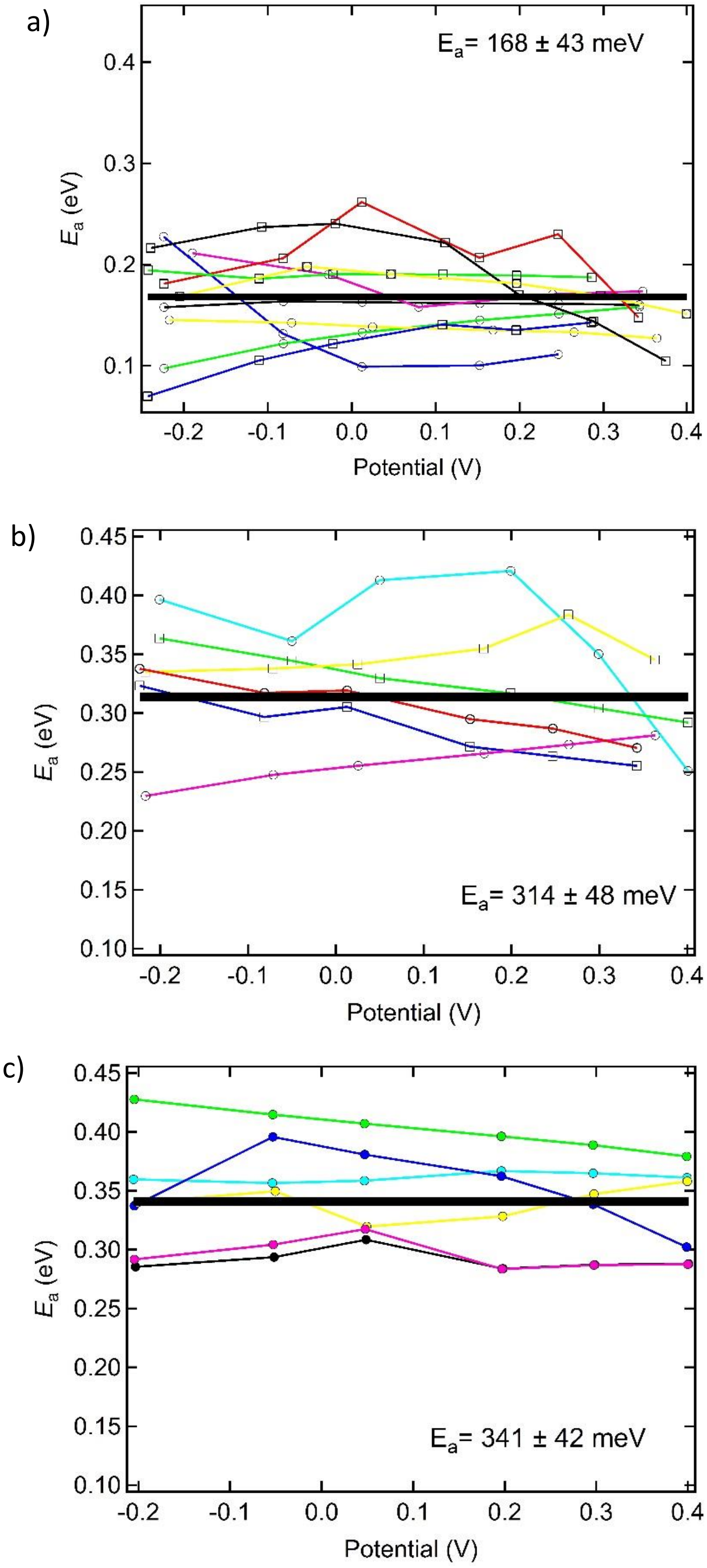

Supplementary Figure 3. Collection of the activation energies for the bromide devices (a) and iodide based devices at high (b) and low light intensity (c). The black line is the average of all measurements.

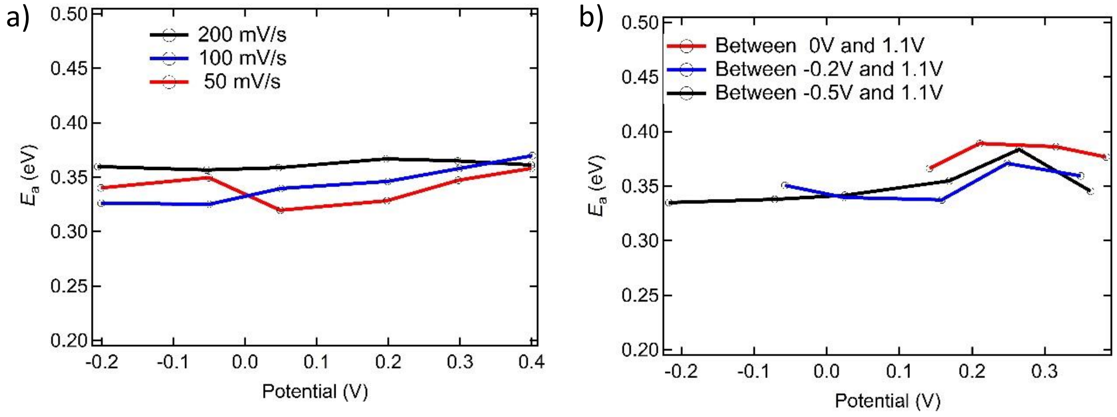

Supplementary Figure 4. Activation energy for the different scan velocities (a) and different scan bounds (b) (for an iodide based device at 1sun light intensity).

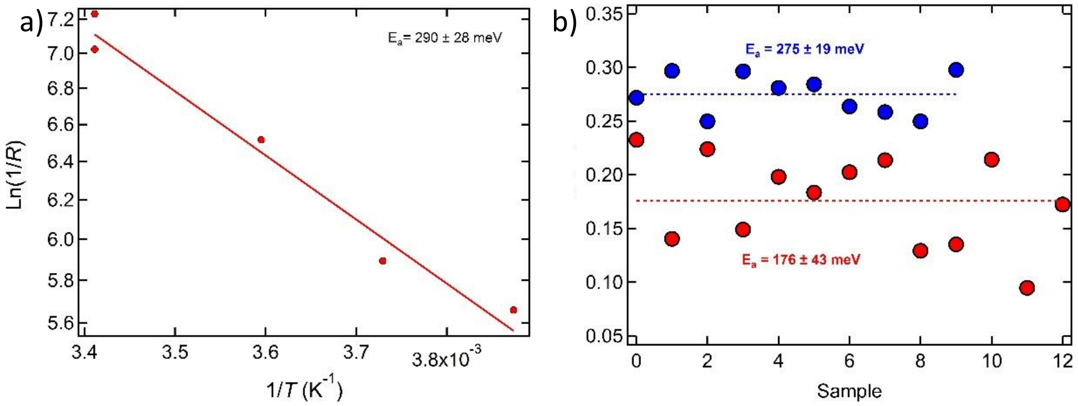

Supplementary Figure 5. a) The slope of the *JV* curve in forward direct at 0V is used to calculate the activation energy treating it similarly to Arrhenius type dependence. It is the same data processed as in Figure 1 and 2 of the main manuscript. b) Activation energies determined from the slope of the forward scan at 0V for the iodide based devices (blue) and for the bromide based devices (red).

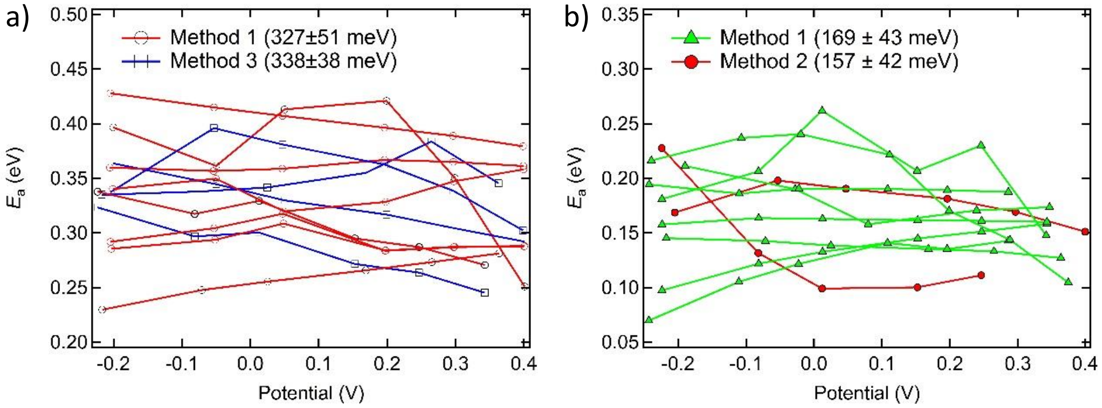

Supplementary Figure 6. Activation energies for the devices made by the fabrication routs 1, 2 and 3 (see supplementary methods for further explanations); a) for the iodide based devices and b) for the bromide based devices.

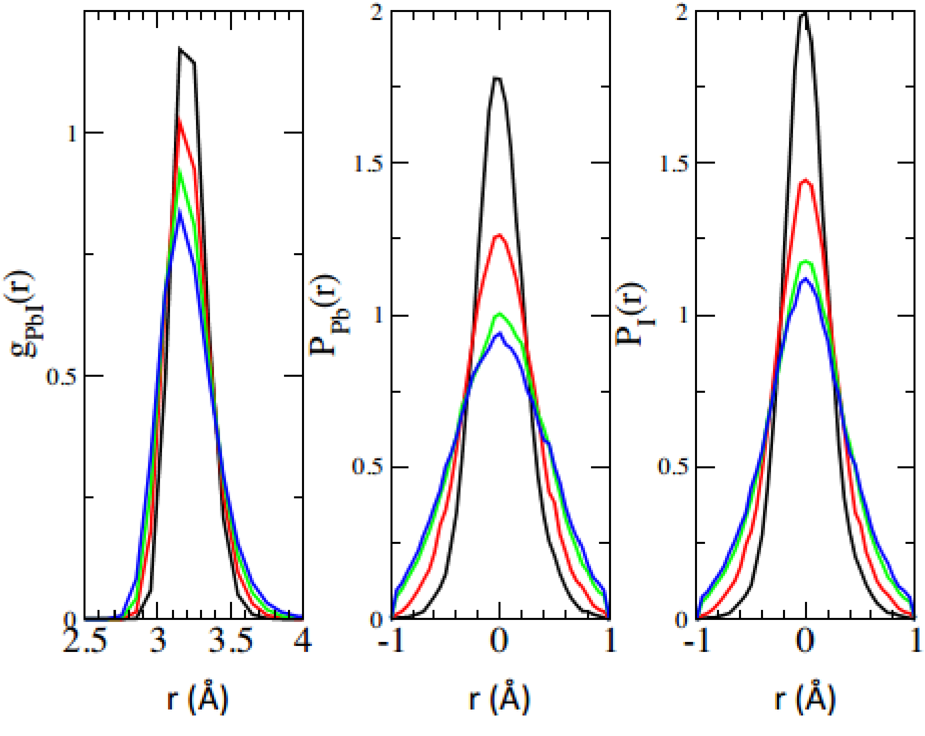

Supplementary Figure 7. (left) Pb-I pair correlation functions relative to Pb-I bonding at 100 K (black), 200 K (red), 300 K (green) and 400 K (blue). No splitting is observed in this range, indicating that there is no break of symmetry in the PbI_3 lattice. At higher temperature the peak shifts to slightly shorter distances, which is due to the phase transition toward a more cubic-like structure in combination with a fixed lattice size. (center and right) Distribution of Pb^{2+} and I^- ions with respect to their equilibrium position. These panels confirm that in the 30 ps simulations Pb^{2+} and I^- ions explore a wide domain of their configuration space, further supporting the hypothesis that the lack of observation of any break of symmetry in the Pb-I lattice is not due to the limited simulation time. The central and right panels also show that the softness of $MAPbI_3$ is mainly ascribable to the large mobility of I^- I ons, which can move significantly out of their equilibrium positions following the libration modes of the PbI_6 octahedra.

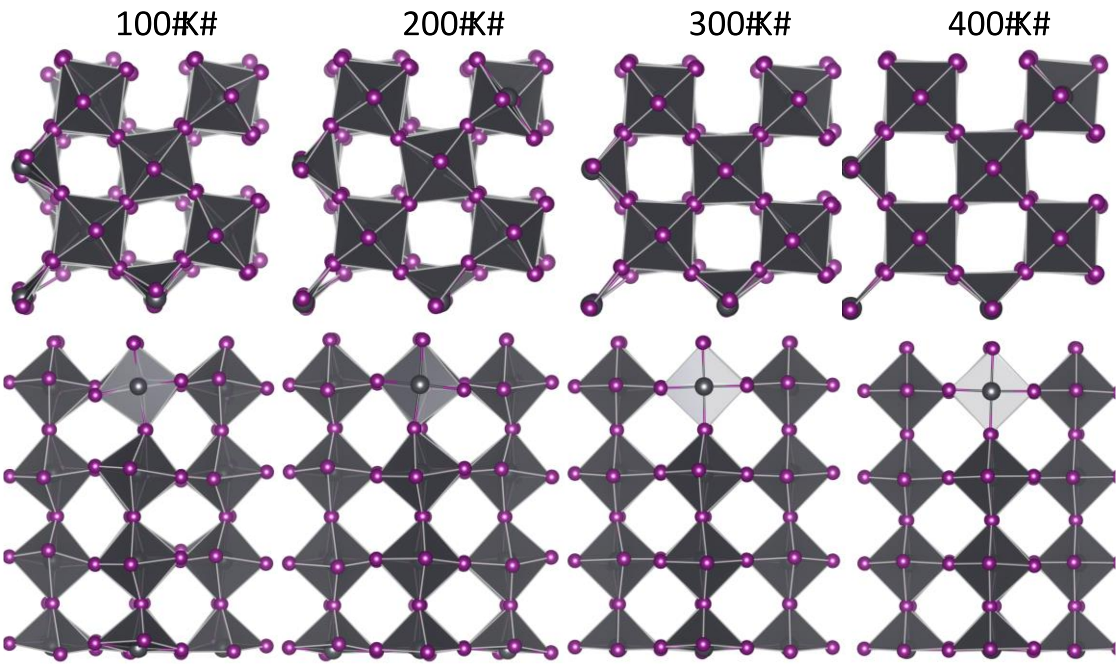

Supplementary Figure 8. Average configuration of the PbI_3 framework computed along the MD simulations at various temperatures. Top view of the PbI_3 framework at each temperature, i.e. the views from the tetragonal axis of the simulation box (top panel). Lateral view, from a convenient direction in the plane orthogonal to the tetragonal axis (bottom panel). The two sets of views have been chosen to better illustrate the effect of temperature on the structure. At 100 K and 200 K the structure shows large tilting angles along all the axes of the PbI_6 octahedra, and the structure is orthorhombic-like. At 300 K these angles are reduced, especially along the axis orthogonal to the tetragonal crystal axis, and the structure is tetragonal-like. Finally, at 400 K, the tilting angles are almost zero, and the structure is cubic-like.

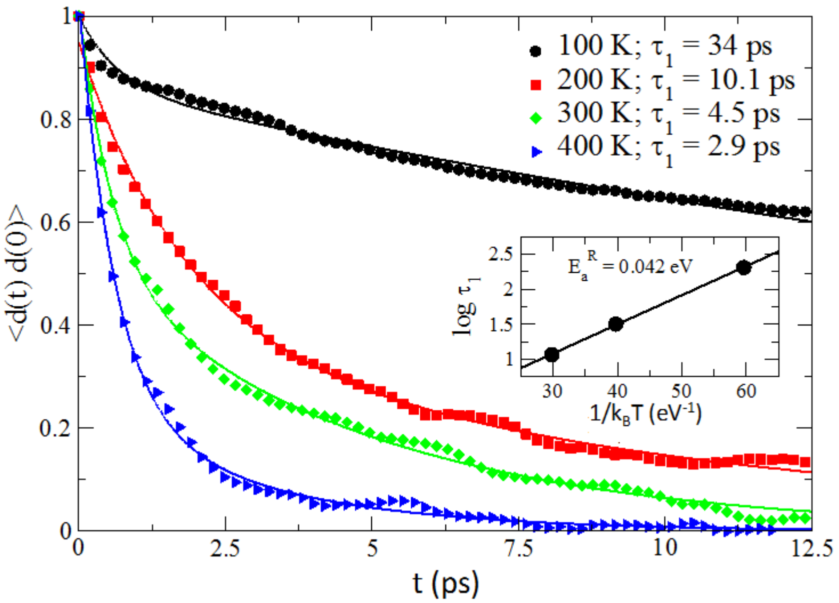

Supplementary Figure 9. Time dependent autocorrelation function of the unit C-N vectors. The autocorrelation function has been computed averaging over the microcanonical ensemble sampled by NVE first-principle MD simulations performed at three different energies corresponding to 100, 200, 300 and 400 K.

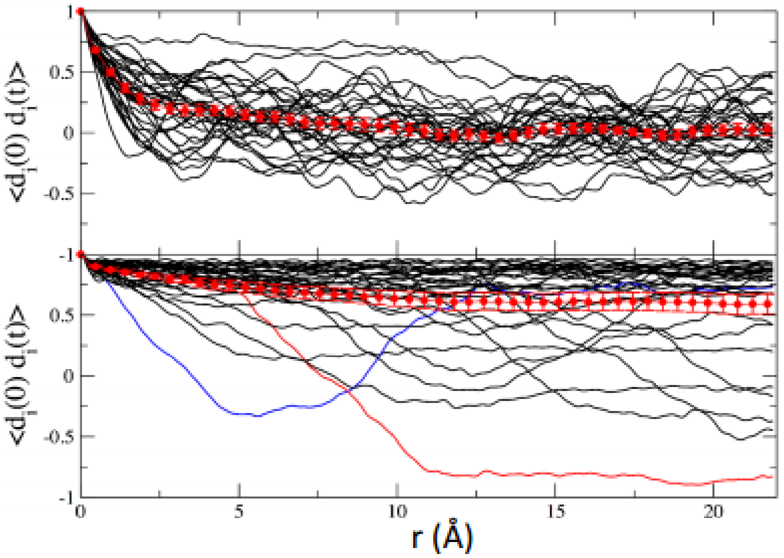

Supplementary Figure 10. the autocorrelation function of individual cations,$\langle \boldsymbol{d_i(0)\cdot d_i(t)} \rangle$, at 300 (top) and 100 K (bottom). In red is reported the total autocorrelation function, $\langle \boldsymbol{d(0)\cdot d(t)} \rangle$, together with the associated error. The error, $\boldsymbol{\Delta}$, is estimated from the standard deviation over the $\langle \boldsymbol{d_i(0)\cdot d_i(t)} \rangle$ under the hypothesis of independent dynamics of the MA cations: $\boldsymbol{\Delta} = \pm\boldsymbol{\sigma}/\sqrt{\boldsymbol{N}}$, with $\boldsymbol{\sigma}$ standard deviation of the autocorrelation function and $\boldsymbol{N}$ number of MA cations.

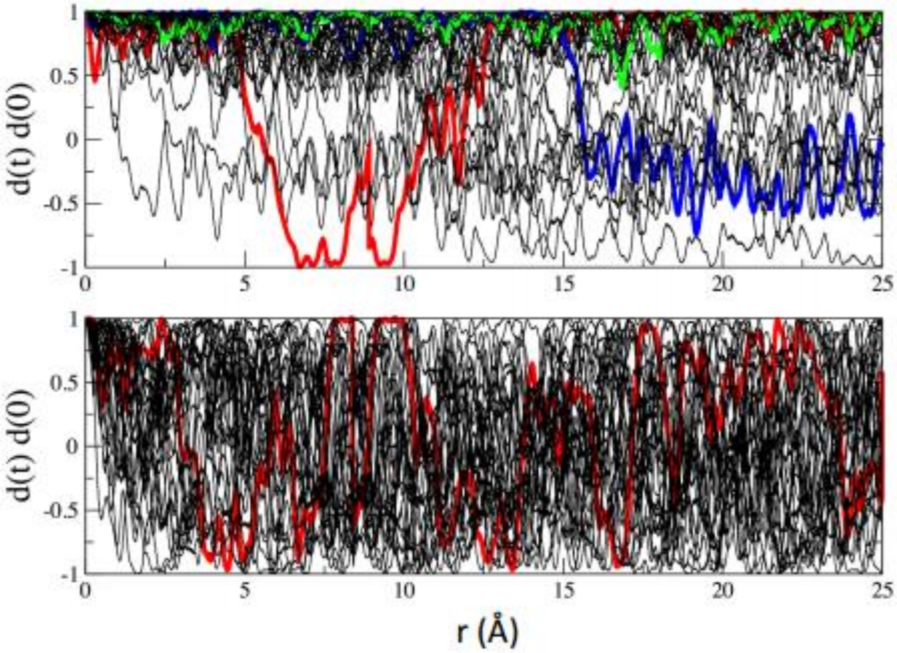

Supplementary Figure 11. Instantaneous correlation function, $\boldsymbol{d_i(0)\cdot d_i(t)}$, as a function of time at 100 (top) and 300 K (bottom). The colored lines in the top panel represent the three typical trajectories observed along the simulation: an MA cation oscillating around its initial orientation (green); an MA cation exploring another metastable state and coming back to its initial state without loosing correlation (red); an MA cation visiting and remaining in a new state, loosing memory of its initial orientation (blue). The red curve in the bottom panel illustrates a typical dynamics of the orientation of an MA cation at 300 K.

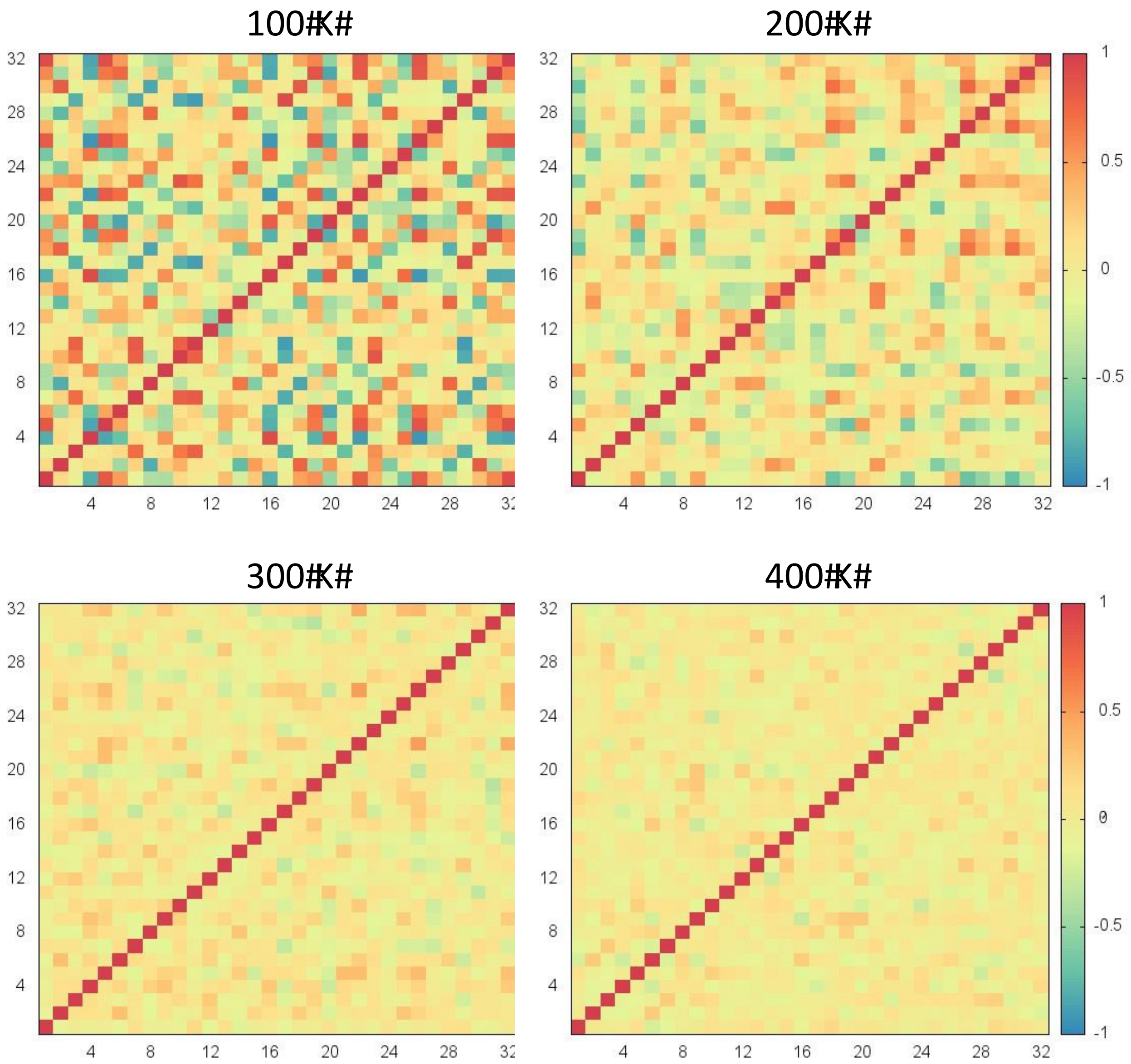

Supplementary Figure 12: Color map of the spatial correlation function. The color of a block in a panel represents the degree of correlation between the orientations of the corresponding pair of MA^+ ions. If the color is red, the two ions are highly correlated, i.e. they tend to have the same orientation. If the color is blue the two ions are highly anticorrelated, that is they tend to have the opposite orientation. If the color is yellow they are uncorrelated, i.e. the orientation of one has no effect on the orientation of the other. The diagonal elements of these panels is the “self”-correlation, which is obviously always 1.

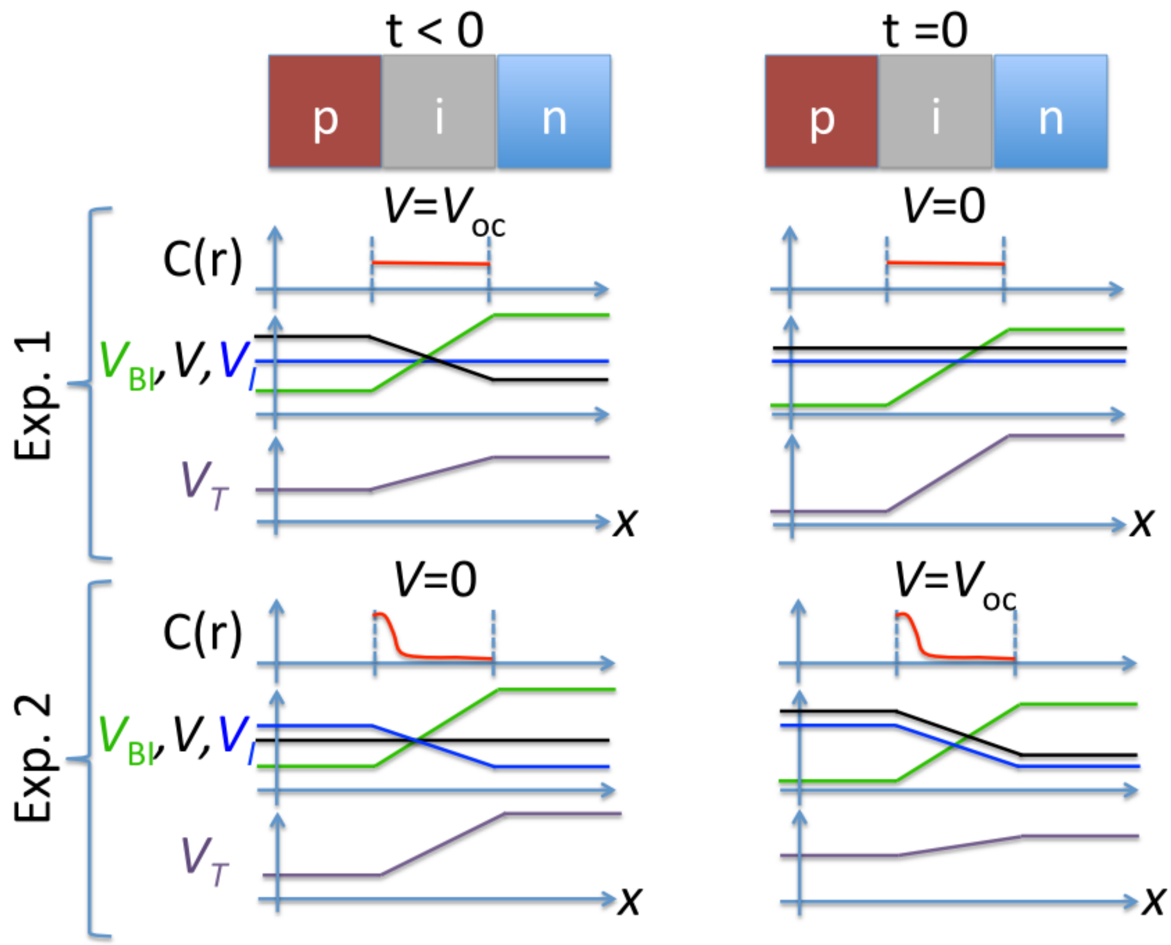

Supplementary Figure 13. Sketch of the effect of the built in plus external bias on the mobile ionic charges, of distributions C(r), before, t<0; and at the switching time, t=0, of the biasing potential, V (black lines). The ionic reaction potential, V_I (blue lines), is sketched in the hypothesis, consistent with experiments and simulation results, that the mobile ionic species is the positive iodide vacancy, V_I°. Finally, the built in potential, V_{BI} (green lines), is added to the other contributions giving the total potential, V_T (purple lines), acting on the charge carriers. The ionic potential distribution (V_I) is assumed to be smooth just for illustrative presentation. Charge localization can give rise to more complex situations, see e.g. [24] .

Supplementary Notes 1

Computational details

First principles molecular dynamics simulations were performed within the framework of the Generalized Gradient Approximation to Density Functional Theory in the Perdew-Burke-Ernzerhof (PBE) formulation.[4] Simulations were performed using the CPMD code.[5] The interaction between valence electrons and nuclei plus core electrons is described by Troullier-Martins pseudopotentials[6]. Kohn-Sham orbitals were expanded in a plane wave basis set with a cutoff of 80Ry. The computational sample consists of a 2x2x2 supercell of the simple tetragonal analogue of the experimental body-centered tetragonal $MAPbI_3$ cell.[7, 8] This simulation box contains 32 stoichiometric units, i.e. 384 atoms. The sample was heated to 100, 200, 300 and 400 K by velocity rescaling. After 2 ps of thermalization the simulation is continued in the microcanonical ensemble for additional 30 ps. Despite the absence of a thermostat, the average temperatures of the three systems remained very close to the target, indicating that the short thermalization time was sufficient.

A 30 ps sampling time is too short to observe and accurately estimate the properties of phenomena like ionic migration discussed in the main text. This, in fact, requires the use of special rare event techniques (see below). Nevertheless, in the following we show that this simulation time is sufficient to investigate possible phenomena related to ferroelectricity.

Within 30 ps the Pb^{2+} and I^- ions move over a wide portion of the configuration space (See Supplementary Figure 7). This is consistent with previous MD simulations,[9] which indicated $MAPbI_3$ to be a structurally soft material. Thus, if a configuration with broken symmetry in the Pb-I bonds is a (meta)stable state of the system, as hypothesized to justify the possible ferroelectricity of $MAPbI_3$,[10] we expect that this state would have been identified in our simulations. Further support to this hypothesis, that our simulations sufficiently investigate the Pb-I configuration space, comes from the fact that, consistent with experiments, we observed the expected phase ordering, within the accuracy of MD simulations, with the temperature: orthorhombic-like at 100 and 200 K, tetragonal-like at 300 K, and cubic-like 400 K (see Supplementary Figure 8). Thus, the fact that the first peak of the Pb-I pair correlation function is not split in two, and the same holds true for the distribution of Pb and I around their equilibrium position (Supplementary Figure 8), indicates that in the 100-400 K range the PbI_3 lattice of $MAPbI_3$ does not present a configuration which might give raise to ferroelectricity in none of the concerned crystalline phases.

We also investigated the alignment of MA molecules as possible source of ferroelectricity. In particular, we computed the time autocorrelation function of the MA orientation (see also discussion in the main text): $\langle \boldsymbol{d}(t) \cdot \boldsymbol{d}(0) \rangle \sim {}^{1}/_{N} \sum_{i=1}^{N} {}^{1}/_{M} \sum_{\alpha=1}^{M} \boldsymbol{d_i}(t + t_\alpha) \cdot \boldsymbol{d_i}(t_\alpha)$. It is worth remarking that the time autocorrelation function, and thus the correlation time, is computed not with respect to the initial configuration of the MD simulations, but as an average over all the pairs of configurations at a given lag time with respect to each other (sum over $\alpha = 1, M$). This amounts to considering each configuration in the MD as a potential initial configuration from which to compute the time autocorrelation function. Thus, the (short) correlation time measured in our

simulations is not due to the specific choice of the initial configuration but is a "genuine" phenomenon.

The $\langle \boldsymbol{d}(t) \cdot \boldsymbol{d}(0) \rangle$ time autocorrelation functions, together with the fitted (bi)exponential curves, are shown in the Supplementary Figure 9. A double decaying exponential was necessary to fit the curves: $\langle \boldsymbol{d}(t) \cdot \boldsymbol{d}(0) \rangle = a_1 e^{-t/\tau_1} + a_2 e^{-t/\tau_2}$. This means that the decorrelation of MA orientation is driven by two phenomena characterized by a shorter and a longer correlation time, $\tau_1 > \tau_2$. We notice that the correlation time of the slower process decreases with temperature, with the correlation time at 300 K nicely matching experimental results.[11, 12]
The dynamics of MA^+ at 100 K deserves special attention. At this temperature the correlation time is on the same order of magnitude as the duration of the simulations. This means that the statistics of the simulation is relatively poor. Indeed, a closer inspection reveals that the dynamics of MA^+ at 100 and 300 K is different. This is better illustrated by the autocorrelation function of individual cations, $\langle \boldsymbol{d_i}(0) \cdot \boldsymbol{d_i}(t) \rangle$ (Supplementary Figure 10), and the corresponding instantaneous function, i.e. the cosine between the orientation of a given MA^+ molecule at time *t* with respect to its orientation at the beginning of the simulation (Supplementary Figure 11). At 300 K the autocorrelation functions of all the MA ions follow an analogous decaying trend. This is because the molecules assume all possible orientations with respect to the initial one (see, for example, the red curve in the Supplementary Figure 11). On the contrary, at 100 K there are MA ions showing a very small decay of $\langle \boldsymbol{d_i}(0) \cdot \boldsymbol{d_i}(t) \rangle$, while others decay quickly but then remain close to a plateau value. This is because some MA^+ cations oscillate around their initial orientation (green curve of Supplementary Figure 11); others visit other (metastable) states and quickly come back oscillating around their initial configuration (red). Finally, some cations visit and get stuck in another state (blue). This is a typical behavior of stochastic, thermally activated, events, which take place in presence of sizeable barriers, i.e. barriers that are higher than the thermal energy of the system, $\boldsymbol{k_B}T$. As discussed in detail below, the barrier for the reorientation of MA^+ is ~ 0.04 eV. This barrier is ~1.5 times the thermal energy at room temperature but is ~5 times the thermal energy at 100 K (neglecting a possible increase of the barrier associated to the transition to the orthorhombic phase). Thus, while the reorientation of MA^+ is fast at room temperature, at low temperatures, in the orthorhombic phase, it is essentially forbidden. This is consistent with recent quasi-elastic neutron scattering experimental results, which show a freezing of the reorientation modes of MA^+ at low temperatures (*T* < 130 K)[13]. Qualitatively analogous results have been obtained by classical MD,[14] confirming that the different behavior at high and low temperatures is not due to size effects.

Taking into account only the relaxation time at 200, 300 and 400 K, i.e. at those temperatures for which there is no long time trapping into metastable states, and the correlation time estimated from the simulations is reliable, we compute the reorientation energy barrier, (see inset of Supplementary Figure 9), $E_a^R \sim 0.04$ eV. This value is in good agreement with several experimental results ($E_a^R \sim 0.1$ eV,[15] ~0.1 eV,[16] ~0.12 eV,[17] and ~0.095 and ~0.07 eV for the cubic and tetragonal phase, respectively[13]). The E_a^R is slightly larger than another experimental result[18] that has appeared recently, (0.009 eV), which is, however, one order of magnitude smaller than previously reported experimental values.

If the dynamics of the MA^+ molecules is uncorrelated, i.e. if there are no domains with a specific orientation, the time for decorrelating the orientation of a single MA^+ ions is of the same order of magnitude as the one for polarizing the sample as a result of MA^+ ions reorientation under the action of an electric field. To test whether the dynamics of MA^+ ions is correlated or not we computed the spatial correlation function, $\langle \boldsymbol{d_i} \cdot \boldsymbol{d_j} \rangle \sim {}^{1}/_{N} \sum_{i=1}^{N} {}^{1}/_{M} \sum_{\alpha=1}^{M} \boldsymbol{d_i}(t_\alpha) \cdot \boldsymbol{d_j}(t_\alpha)$. Color maps of $\langle \boldsymbol{d_i} \cdot \boldsymbol{d_j} \rangle$ at the four temperatures are shown in the Supplementary Figure 12. Results show that positive and negative (anti) correlation is sizeable at 100 K, but quickly decreases with temperature, becoming very small at room temperature and above, i.e. in the temperature range of interest for the present article. This is consistent with the short reorientation time observed in the experiments [13, 18].

Considering the small correlation between MA^+ ions at room temperature, we conclude that the polarization time of the perovskite layer due to MA^+ reorientation is of the order of ps at 300-400 K. This timescale is very short with respect to the scanning time of even the fastest *JV* measurements, which proves that the origin of the hysteresis of the *JV* curves cannot be the induced alignment of the dipole moment of MA^+ ions.

The very short correlation time for the MA^+ reorientation process may also explain the lack of symmetry breaking of the Pb-I lattice. Frost *et al.*[19] have proposed that the alignment of MA^+ cations might trigger a break of symmetry in the PbI_3 framework. However, the fast change of orientation of MA^+ cations results in a spherically symmetric average force on the framework. Thus, the MA^+ related mechanism for inducing a ferroelectric state is not viable.

The MD simulations of defect containing systems, i.e. of samples with one I^-, Pb^{2+} or MA^+ vacancy, showed no sign of ionic migration on the 30 ps timescale of their duration. This is not surprising because the migration of an ion from of its original site into an empty neighbor one typically requires to overcome an energetic barrier sizeably higher than the thermal energy available to the system, and it is, thus, a rare event (the probability for this event to take place is proportional to $e^{-\mathrm{Ea}/k_BT}$, with k_B Boltzmann constant and k_BT thermal energy). To deal with these cases, one needs to use special simulation techniques. Here we use the string method.[20] The objective of the string method is to identify the most probable path connecting reactants and products. In the present case, reactants and products are two states of our system in which the vacancy of one ionic species, either X^-, Pb^{2+} or A^+, is on one lattice site or has migrated on a neighbor (initially empty) site. The string path is a parametric curve, $\boldsymbol{r}(\lambda)$, in the space of the atomic configurations ($\boldsymbol{r}$ is the 3N vector of the atomic positions). λ is a parameter measuring the degree of progress of the vacancy migration: $\lambda = 0$ when the vacancy is in the initial site, and $\lambda = 1$ when it is in the final site. It can be shown[20] that the most probable path is the one with zero component of the atomic forces in the direction orthogonal to the path:

$$\left[-\boldsymbol{\nabla V}\big(\boldsymbol{r}(\lambda)\big)\right]_{\perp \boldsymbol{r}(\lambda)} = 0$$

In the string method the continuous path $\boldsymbol{r}(\lambda)$ is discretized into a finite number, $\boldsymbol{L}$, of configurations (snapshots), $\{\boldsymbol{r}(\lambda_i)\}_{\boldsymbol{i=1,L}}$. These snapshots satisfy the additional constraint to be at a constant distance from each other: $|\boldsymbol{r}(\lambda_i) - \boldsymbol{r}(\lambda_{i+1})| = |\boldsymbol{r}(\lambda_j) - \boldsymbol{r}(\lambda_{j+1})|$. An initial guess path can be optimized evolving it by a fictitious first order dynamics (steepest descent

"dynamics") in which at each time step each snapshot, $\boldsymbol{r}(\lambda_i)$, is evolved following the component of the atomic forces orthogonal to the path, $-\boldsymbol{\nabla V}\big(\boldsymbol{r}(\lambda_i)\big)(\mathbf{1}-\alpha(\boldsymbol{r}(\lambda_i))\otimes\alpha(\boldsymbol{r}(\lambda_i)))$, where $\mathbf{1}$ is the identity matrix, $\alpha(\boldsymbol{r}(\lambda_i))$ is the tangent to the path at the *i*-th point, and thus $(\mathbf{1}-\alpha(\boldsymbol{r}(\lambda_i))\otimes\alpha(\boldsymbol{r}(\lambda_i)))$ is projector on the plane orthogonal to the path at the point $\boldsymbol{r}(\lambda_i)$. This algorithm, however, is not very efficient as determining the tangent to a discrete approximation to a continuous curve is prone to large numerical errors. In the string method it is recognized that the tangential component of the force has the only effect of altering the condition of equidistance between successive images. Thus, instead of projecting the forces $-\boldsymbol{\nabla V}\big(\boldsymbol{r}(\lambda_i)\big)$ on the plane orthogonal to the path, the snapshots are first evolved according to the entire force, then the equidistance among the snapshots is restored. This second step is performed by moving the snapshots along a locally cubic spline interpolation to the path. This renormalization step, by removing the effect of the tangential component of the force, is formally equivalent to applying the projection. In practice, for each vacancy migration channel, we started from a guess path corresponding to the linear interpolation of the configurations corresponding to the system in the initial and final states. This path is discretized in 20 snapshots. For each step of the string fictitious dynamics we compute the atomic forces using the Generalized Gradient Approximation to the Density Functional Theory in the Perdew-Burke-Ernzerhof (PBE) formulation, like in the case of first-principle MD simulations. The atoms of each image are evolved by one step along the steepest descent dynamics and, then, the string is renormalized. This procedure is repeated till convergence, i.e. until the residual estimated orthogonal force is below a prescribed threshold, and the maximum displacement of the string between two successive iterations is below another prescribed threshold. On the converged string we apply the "climbing algorithm", which moves the higher in energy snapshot upward, toward the maximum of the energy along the string. This allows to accurately determining the energy of the transition state and, from its difference with the energy of the initial and final states, the barrier of the forward and backward vacancy migration processes.

String calculations where performed using the *neb.x* module of the Quantum Espresso package.[21] In this case we used ultrasoft pseudopotentials with a 45 Ry cutoff of the kinetic energy of the planewave basis set. The Brillouin zone is sampled with a 4x4x4 shifted Monkhorst-Pack[20] grid. To validate the accuracy of our computational approach and setup we compared the activation energy for the bromide migration in $CsPbBr_3$ with experimental results**.** The difference between theoretical (270 meV) and experimental barriers (250 meV),[22] is smaller than the DFT accuracy.

Supplementary Notes 2

Mesoscopic model of ion diffusion in perovskite solar cells

In this section we present a model describing the dynamics of ions during the voltage scan. We refer to this model as "mesoscopic" to distinguish it from the microscopic, atomistic, model for ionic migration obtained from atomistic simulations. The present model is based on a continuum description of matter. Thus, ions are described in terms of the associated concentration, $\mathrm{C(r)}$. To make the description of the model clearer, we present it through two thought experiments. Consistent with the description given in Ref. [23], perovskite solar cells are idealized as a p-i-n

junction. In this simplified model we neglect important aspects of real solar cells as, for example, blocking layers. These important aspects require additional, *ad hoc*, investigations that will be discussed in future work.

In p-i-n junctions, like in p-n junctions, the non-uniform carrier distribution can be associated to a potential that varies along the junction, the so-called built in potential, V_{BI}. The external bias, V, together with the built-in potential produces the total potential determining the charge carrier distribution. The field associated to the combined electric potential represents drift forces acting on the mobile ions. This drift force can produce a polarization of the distribution of the mobile ionic species that, then, produces an additional electric potential, V_I, affecting the electronic distribution. Thus, in principle, one should deal with the time dependent and stationary state of the charge carriers and mobile ions in a coupled way. Here, exploiting the separation of the timescale in the evolution of (fast) charge carriers and (relatively slow) mobile ionic species, we describe the behavior of the system introduced above assuming that the fast species are always at the stationary condition consistent with the ionic distribution.

Let us now perform the two thought experiments mentioned above. These thought experiments are summarized in Supplementary Figure 13.

First thought experiment. We prepare the system at $V = V_{oc}$ and let it reach the stationary state. At $V = V_{oc}$ the combined built in and external bias electric field, and thus the drift force, is at the minimum in the range of bias of interest for solar cells applications. In these conditions, the polarization of ions is minimal.

At time t=0 the external bias is instantaneously switched from V_{oc} to 0. The combined potential acting on the charge carriers and the ions is at its maximum. Ions evolve in time according to the drift equation with diffusion

$$j = \mu CF - D\nabla C$$

where μ is the mobility and D the diffusion coefficient of the mobile ionic species. Since we assume ions blocking contacts and no faradaic reaction at the n and p contacts, the drift force, F, tends to accumulate them at the corresponding contacts, depending on their charge. The diffusion *force* tends to restore a uniform distribution of ions in the sample. Thus, initially, when the ionic polarization is at its minimum, only the drift force acts on the sample, till ∇C is large enough that the diffusive contribution is no longer negligible.

According to the above picture, along the backward scan the *JV* characteristics is initially analogous to the ideal case. Then, at longer times the onset of the ionic polarization might results in departure from the ideal p-i-n junction. If we let the system evolve further at $V = 0$, the system reaches another stationary state at high ionic polarization of the perovskite layer.

Second thought experiment. Here we perform the opposite experiment. The system is prepared at $V = 0$, where it reaches the highly polarized stationary state. The external bias is instantaneously switched to V_{oc}. In this condition the drift force is even lower (and possibly slightly inverted) than at the starting point of Exp. 1. The system relaxes to the almost uniform distribution mainly under the action of the diffusion *force*. Of course, the diffusion is not instantaneous. Thus, the *JV* characteristics of the solar cells are different from those of the initial

state of the first thought experiment. In particular, since mobile ions are distributed under the effect of the built in and external potential analogously to the corresponding charge carriers, positive iodide vacancies (V_I°) accumulate at the anode. Before the ions relax to the stationary state at V_{oc} their non-uniform distribution produces a potential term adding up to the external bias. This additional term can give rise to the observed effect in the solar cell devices investigated in this publication. Analogous effects have been demonstrated in the piezophototronic solar cell devices.[24]

Supplementary Methods

Several different types of $MAPbI_3$ and $MAPbBr_3$ samples have been used to estimate the temperature dependence of the observed hysteretic effect.

Device production 1:

Perovskite solar cell devices were prepared on conductive fluorine-doped tin oxide (FTO) coated glass substrates (Tec15, Pilkington). A compact titanium dioxide layer of about 20 nm was deposited by spray pyrolysis of 9 ml ethanol solution containing 0.6 mL titanium diisopropoxide bis(acetylacetonate) (75% in 2-propanol, Sigma-Aldrich) and 0.4mL acetylacetone (≥99%, Sigma-Aldrich) at 450 C in air. On top of this layer, a 300 nm-thick mesoporous titanium dioxide was formed by spin-coating 30 nm sized TiO_2 nanoparticles (30NRT, Dyesol) diluted in ethanol (≥99.8%, Sigma-Aldrich) (1:3.5 w/w) at 4800 r.p.m. for 20 s. The formed layer was gradually heated up to 500 degrees and sintered for 1 hour in oxygen atmosphere. Afterwards, stock solutions of lead iodide (PbI_2) (1,2 M) (99%, Acros) and lead bromide ($PbBr_2$) (1.0 M) (98%, Sigma-Aldrich/99.999%, Sigma Aldrich) were prepared by dissolving in N,N-dimethylformamide (99.8%, Acros) and spin coated on the top of mesoporous TiO_2 layer at 6500 r.p.m. for 20 s. The formed layers were then left for drying for 15 min on the hotplate at 85 degrees. The depositions of lead salts were performed two times. Subsequently, on the top of that layer, 0.05 M CH_3NH_3I (for lead iodide films synthesized after Im et al.[1]) or CH_3NH_3Br ethanol solution was sprayed and left for 20 s before spin coating at 3000 r.p.m. for 30 s. The synthesis of CH_3NH_3I and CH_3NH_3Br was conducted as described by Etgar et al.[2] The formed films were then left for drying for 15 min at 85 degrees. On the top of formed perovskite films, a hole transporting material (HTM) was deposited by spin coating at 3000 r.p.m. for 20 s. HTM was prepared by dissolving 74 mg spiro-MeOTAD in 1 ml chlorobenzene and additionally mixing it with 28.8 µL of tert-butylpyridine (96%, Sigma-Aldrich), 17.5 µL of bis(trifluoromethane)sulfonimide lithium salt (LiTFSI) (99.95% Sigma-Aldrich) (stock solution Li-TFSI 520 $mg\cdot mL^{-1}$ in acetonitrile (99.9%, Acros)) and 29 µl of tris(2-(1H-pyrazol-1-yl)-4-tert-butylpyridine)cobalt(III)[3] (stock solution FK-209 300 $mg\cdot mL^{-1}$ in acetonitrile). Devices were finally completed by thermally evaporating 60 nm thick gold layer on the top of the HTM.

Device production 2:

FTO glass was cleaned in an ultrasonic bath with 2% Hellmanex detergent for 30 minutes and IPA for 15 minutes, followed by rinsing with acetone and an oxygen plasma treatment for 15 minutes. A compact TiO_2 was deposited via spray pyrolysis at 450°C from a precursor solution of titanium

diisopropoxide bis(acetylacetonate) in anhydrous ethanol. The samples were kept at 450°C for 20 minutes and then gradually cooled down to room temperature. A mesoporous TiO2 layer was deposited by spin-coating at 2000 rpm (ramp: 2000 rpm s-1) for 60 s using the commercially available 18NRT Dyesol paste (diluted with ethanol in a 2:7 weight ratio). The film was annealed at 100°C for 10 minutes, then gradually heated to 500 °C for 30 min, and subsequently left to cool to room temperature. The MAPbBr3 films were deposited in a nitrogen filled glove box following a 2 step procedure. In the first step a 1M $PbBr_2$ solution (in DMF) was spin-coated on mesoporous titania at 2500 rpm (ramp: 2500 rpm s-1) for 60 s, followed by annealing at 70 °C for 30 min. In the second step, the films were exposed to MABr vapor. For this, we dispersed MABr powder on the bottom of a glass jar that was closed by a tightly fitting lid on which we mounted the substrates such that the $PbBr_2$ treated side faced the MABr powder. The glass dish was put on a hot plate at 150 °C for 10 minutes, and then rinsed in IPA at room temperature. The hole transporting material made of 59 mM Spiro-OMeTAD in chlorobenzene with added 330 mol% tBP and 50 mol% of Li-TFSI from a 1.8M stock solution in ACN was deposited by spin-coating at 2000 rpm (ramp: 2000 rpm s-1) for 60 s. Finally, 80 nm thick gold electrodes were evaporated onto the devices through a shadow mask.

Device production 3

Chemically etched FTO glass (NSG 10Ω, Nippon Sheet Glass) was sequentially cleaned with detergent solution, deionized water, acetone and ethanol under ultrasonication for 30 min. A 30 nm-thick TiO_2 blocking layer was deposited with diluted titanium diisopropoxide bis(acetylacetonate) solution (Sigma-Aldrich) in ethanol by spray pyrolysis at 450℃. 350 nm-thick mesoporous TiO_2 layers with Dyesol 18-NRT paste (particle size: ~20 nm; diluted with ethanol, 1:3.5 weight ratio) was made by spin coating at 5,000 rpm for 30 s and heating at 500℃ for 30 min to burn organic components. The mesoporous TiO_2 film was immersed in 0.02 M aqueous $TiCl_4$ (>98%, Aldrich) solution at 70°C for 30 min. After washing with DI water and drying, the film was heated at 500°C for 30 min. Mesoporous film thickness is about 300 to 400 nm. For the deposition of methylammonium lead iodide, 1.0 M of lead iodide solution in N,N-dimethylformamide kept at 70℃ was firstly spin coated at 6,500 rpm for 30 sec on mp-TiO2 electrode and then dried at 70℃ for 15 min. After cooling to room temperature, the film was immersed in a solution of methylammonium iodide in isopropanol (8 mg ml^{-1}) for 20 s, shortly rinsed with isopropanol and dried again at 70℃ for 15 min. The HTM solution was prepared by dissolving 72.3 mg of (2,2`,7,7`-tetrakis(N,N-di-p-methoxyphenylamine)-9,9-spirobifluorene) (spiro-MeOTAD), 28.8 uL of 4-tert-butylpyridine (TBP, Aldrich), 17.5 uL of a stock solution of 520 mg/mL of lithium bis(trifluoromethylsulphonyl)imide in acetonitrile and 29 uL of a stock solution of 300 mg/mL of tris(2-(1H-pyrazol-1-yl)-4-tert-butylpyridine)cobalt(III) bis(trifluoromethylsulphonyl)imide in acetonitrile in 1 mL of chlorobenzene. This solution was spin-coated at 4,000 rpm for 30 s before deposition of 60 nm-thick gold counter electrodes by evaporation method.

JV curve measurements

The measurements were performed with a Bio-Logic SP-300. As white light source we used a LED array. For temperature control we used a climatic chamber from Angelantoni (ST16). The measurements of the *JV* curve were performed after the device had equilibrated 10 minutes at

the desired temperature. To minimize changes of the devices' photovoltaic performance during the measurements they were subjected to about 30min "aging" at 1sun before the temperature measurements were started.

The efficiency of the devices with iodide was in the range of 10 to 14% PCE. In the case of the bromide devices the lower efficiency is mainly due to the low J_{sc} (about 3 to 5 mA/cm^2), resulting in ~ 2 to 4% efficiency. Since the light intensity dependence can impact the hysteretic behavior we have measured the iodide based devices also at low light intensities yielding a similar J_{sc} like the $MAPbBr_3$ devices. The hysteresis effect of the devices with bromide is generally less pronounced, which also leads to a higher error in the estimation of the activation energy from experimental data (as can be seen in the standard deviation).

For further detail on the measurements protocol and data analysis see the experimental results part.

Supplementary References

1. Kim H-S*, et al.* Lead Iodide Perovskite Sensitized All-Solid-State Submicron Thin Film Mesoscopic Solar Cell with Efficiency Exceeding 9%. *Scientific Reports* **2**, (2012).

2. Etgar L*, et al.* Mesoscopic CH3NH3PbI3/TiO2 Heterojunction Solar Cells. *J Am Chem Soc* **134**, 17396-17399 (2012).

3. Burschka J, Kessler F, Nazeeruddin MK, Grätzel M. Co(III) Complexes as p-Dopants in Solid-State Dye-Sensitized Solar Cells. *Chem Mater* **25**, 2986-2990 (2013).

4. Xiao Z*, et al.* Giant switchable photovoltaic effect in organometal trihalide perovskite devices. *Nat Mater* **14**, 193-198 (2015).

5. CPMD, http://www.cpmd.org/. MPI für Festkörperforchung Stuttgart and IBM Corp.

6. Troullier N, Martins JL. Efficient pseudopotentials for plane-wave calculations. *Phys Rev B* **43**, 1993-2006 (1991).

7. Onoda-Yamamuro N, Matsuo T, Suga H. Calorimetric and IR spectroscopic studies of phase transitions in methylammonium trihalogenoplumbates (II)†. *J Phys Chem Solids* **51**, 1383-1395 (1990).

8. Stoumpos CC, Malliakas CD, Kanatzidis MG. Semiconducting tin and lead iodide perovskites with organic cations: Phase transitions, high mobilities, and near-infrared photoluminescent properties. *Inorg Chem* **52**, 9019-9038 (2013).

9. Frost JM, Butler KT, Walsh A. Molecular ferroelectric contributions to anomalous hysteresis in hybrid perovskite solar cells. *Apl Mater* **2**, 081506 (2014).

10. Zhao Y*, et al.* Anomalously large interface charge in polarity-switchable photovoltaic devices: an indication of mobile ions in organic-inorganic halide perovskites. *Energy Environ Sci*, 1256-1260 (2015).

11. Poglitsch A, Weber D. Dynamic Disorder in Methylammoniumtrihalogenoplumbates(Ii) Observed by Millimeter-Wave Spectroscopy. *J Chem Phys* **87**, 6373-6378 (1987).

12. Jamnik J, Maier J. Treatment of the impedance of mixed conductors - Equivalent circuit model and explicit approximate solutions. *J Electrochem Soc* **146**, 4183-4188 (1999).

13. Chen T*, et al.* Rotational dynamics and its relation to the photovoltaic effect of CH3NH3PbI3 perovskite. Preprint at http://adsabs.harvard.edu/abs/2015arXiv150602205C (2015).

14. Mattoni A, Filippetti A, Saba MI, Delugas P. Methylammonium Rotational Dynamics in Lead Halide Perovskite by Classical Molecular Dynamics: The Role of Temperature. *J Phys Chem C* **119**, 17421-17428 (2015).

15. Wasylishen RE, Knop O, Macdonald JB. Cation rotation in methylammonium lead halides. *Solid State Commun* **56**, 581-582 (1985).

16. Onoda-Yamamuro N, Matsuo T, Suga H. Dielectric study of CH3NH3PbX3 (X = Cl, Br, I). *J Phys Chem Solids* **53**, 935-939 (1992).

17. Mosconi E, Quarti C, Ivanovska T, Ruani G, De Angelis F. Structural and electronic properties of organo-halide lead perovskites: a combined IR-spectroscopy and ab initio molecular dynamics investigation. *Phys Chem Chem Phys* **16**, 16137-16144 (2014).

18. Leguy AMA*, et al.* The dynamics of methylammonium ions in hybrid organic-inorganic perovskite solar cells. *Nat Commun* **6**, (2015).

19. Frost JM, Butler KT, Brivio F, Hendon CH, van Schilfgaarde M, Walsh A. Atomistic Origins of High-Performance in Hybrid Halide Perovskite Solar Cells. *Nano Lett* **14**, 2584-2590 (2014).

20. E W, Ren W, Vanden-Eijnden E. Simplified and improved string method for computing the minimum energy paths in barrier-crossing events. *J Chem Phys* **126**, 164103 (2007).

21. Giannozzi P*, et al.* QUANTUM ESPRESSO: a modular and open-source software project for quantum simulations of materials. *J Phys: Condens Matter* **21**, 395502 (2009).

22. Mizusaki J, Arai K, Fueki K. Ionic conduction of the perovskite-type halides. *Solid State Ionics* **11**, 203-211 (1983).

23. Tress W, Marinova N, Moehl T, Zakeeruddin SM, Mohammad K N, Gratzel M. Understanding the rate-dependent J-V hysteresis, slow time component, and aging in CH3NH3PbI3 perovskite solar cells: the role of a compensated electric field. *Energy Environ Sci*, 995-1004 (2015).

24. Wang ZL. Piezotronics and Piezo-Phototronics. In: *Microtechnology and MEMS*. Springer Berlin Heidelberg (2012).
